# Defining durum wheat ideotypes adapted to Mediterranean environments through remote sensing traits

**DOI:** 10.3389/fpls.2023.1254301

**Published:** 2023-09-05

**Authors:** Adrian Gracia-Romero, Thomas Vatter, Shawn C. Kefauver, Fatima Zahra Rezzouk, Joel Segarra, María Teresa Nieto-Taladriz, Nieves Aparicio, José Luis Araus

**Affiliations:** ^1^ Integrative Crop Ecophysiology Group, Plant Physiology Section, Faculty of Biology, University of Barcelona, Barcelona, Spain and AGROTECNIO (Center for Research in Agrotechnology), Lleida, Spain; ^2^ Instituto Nacional de Investigación y Tecnología Agraria y Alimentaria (INIA), Madrid, Spain; ^3^ Agro-technological Institute of Castilla y León (ITACyL), Valladolid, Spain

**Keywords:** vegetation indices, canopy temperature, carbon isotope composition, highthroughput phenotyping, UAV

## Abstract

An acceleration of the genetic advances of durum wheat, as a major crop for the Mediterranean region, is required, but phenotyping still represents a bottleneck for breeding. This study aims to define durum wheat ideotypes under Mediterranean conditions by selecting the most suitable phenotypic remote sensing traits among different ones informing on characteristics related with leaf pigments/photosynthetic status, crop water status, and crop growth/green biomass. A set of 24 post–green revolution durum wheat cultivars were assessed in a wide set of 19 environments, accounted as the specific combinations of a range of latitudes in Spain, under different management conditions (water regimes and planting dates), through 3 consecutive years. Thus, red–green–blue and multispectral derived vegetation indices and canopy temperature were evaluated at anthesis and grain filling. The potential of the assessed remote sensing parameters alone and all combined as grain yield (GY) predictors was evaluated through random forest regression models performed for each environment and phenological stage. Biomass and plot greenness indicators consistently proved to be reliable GY predictors in all of the environments tested for both phenological stages. For the lowest-yielding environment, the contribution of water status measurements was higher during anthesis, whereas, for the highest-yielding environments, better predictions were reported during grain filling. Remote sensing traits measured during the grain filling and informing on pigment content and photosynthetic capacity were highlighted under the environments with warmer conditions, as the late-planting treatments. Overall, canopy greenness indicators were reported as the highest correlated traits for most of the environments and regardless of the phenological moment assessed. The addition of carbon isotope composition of mature kernels was attempted to increase the accuracies, but only a few were slightly benefited, as differences in water status among cultivars were already accounted by the measurement of canopy temperature.

## 1 Introduction

The resilience of staple food crops like wheat to unfavorable climatic conditions plays a vital role in ensuring food security. This is particularly evident for durum wheat, one of the main herbaceous crops in the Mediterranean, which is frequently subjected to abiotic stresses such as water stress and high temperatures that limit its productivity. Moreover, in comparison with bread wheat, the genetic advance of durum wheat, at least in the Mediterranean basin, has been rather modest (Acreche et al., 2008; Chairi et al., 2018; Chairi et al., 2020a; Chairi et al., 2020b). Even at the European level, there is the perception that current breeding programs and cultivar selection practices do not sufficiently prepare for climatic uncertainty and variability (Kahiluoto et al., 2019). Understanding the complex interactions between the genotype, the environmental conditions, and the specific agronomic and management (G × E × M) conditions is crucial for the success of crop improvement programs. Because crop performance, understood as grain yield (GY) response to the environment, is a complex trait, breeding selection has to include a wide range of environmental growing conditions. Thus, for durum wheat in the Mediterranean basin, a strong association between the local climate and the yield formation strategies of the cultivars has been reported (Kyratzis et al., 2022; Royo et al., 2022). In fact, multi-environment evaluation has been traditionally considered as a key strategy in breeding programs to boost yield and in maintaining stability (Reynolds et al., 2009; Watt et al., 2020). This includes not only characterizing the target trait, which is usually the GY, and defining the targeted growing conditions but also accounting for diverse crop factors such as phenology and other morphophysiological traits putatively associated with yield (Araus et al., 2008).

The term “ideotype” defines the combination of morphological and physiological traits that theoretically optimize crop performance under a particular environmental condition. The first wheat ideotype was proposed by Donald (1968) for non-limiting agronomic conditions and was defined as short stature plants with, strong stems, low tillering capacity, and large erect ears. Accordingly, the breeding of new cultivars was focused on an improvement in lodging prevention, which was amenable to high nitrogen fertilizer inputs (Hamblin, 1993). An earlier progression to the stages of heading, anthesis, maturity, and, to a lesser extent, early vigor has been recurrent traits when designing “Mediterranean ecotypes” (Loss and Siddique, 1994; Sadras and Richards, 2014). However, the benefits from exploring shorter crop cycles seem to have been virtually exhausted (Chairi et al., 2018), so other traits conferring adaptation to Mediterranean conditions must be explored. Because wheat is basically grown under rainfed conditions and, because of the climate change, drought episodes are expected to be more common and more severe (Al-Khayri et al., 2019; Zampieri et al., 2020), the selection criteria that will determine GY should be focused on adaptation traits for increased temperatures and water stress (Zampieri et al., 2017).

The concept of crop ideotype allows breeders to focus their selection process on a specific trait-based model, rather than just the selection for yield. However, the concept of ideotype needs to progress even further from the traditional characterization (i.e., visual scoring or destructive sampling) of traits putatively associated with crop performance, by exploiting the current developments in high-throughput phenotyping to assess those same traits (Reynolds et al., 2009), including statistical models (Paleari et al., 2020). Modern breeding strategies are moving away from the development of high-plasticity genotypes for enhanced performance under a wide range of environments, toward modeling a specific set of genotype characteristics for a particular environmental growing condition (Jaradat, 2018). Therefore, the more modern concept of ideotype can be defined as seeking the best crop phenotype to grow in a given environment within a defined cropping system (Martre et al., 2015). Thus, Padovan et al. (2020) used simulation crop models to define cultivar selection strategies in durum wheat based on higher leaf area index and radiation use efficiency parameters for cooler and wetter locations, whereas short-cycle cultivars with high grain dry matter potential were preferred for hotter and dryer locations.

Establishing an ideotype design for a target environment may be also developed empirically through phenotyping, which implies the existence of concrete guidelines throughout the crop cycle for traits that determine yield. To that end, one of the main objectives of crop phenotyping is to identify and quantify a key set of traits that will determine crop growth and agronomic performance in terms of yield and define how and when to measure them (Watt et al., 2020). Plant phenotyping pursues the characterization of genotypes as they interact with the environment, and studies are underway to develop high-throughput plant phenotyping (HTPP) methodologies at affordable costs, which is an issue that has often been regarded as a major bottleneck in the breeding process (Araus and Cairns, 2014). Thus, once ideotypes are established, HTPP will help to recognize genotypes exhibiting these ideotypic traits among large germplasm sets. Broadly, HTPP is currently mostly based on non-destructive evaluations mainly of a remote sensing nature at different levels, from measurements in single leaves such as pigment content or chlorophyll fluorescence to the ever more frequent evaluation at the canopy level using different types of sensors either from the ground or placed on aerial platforms (Furbank and Tester, 2011; Fiorani and Schurr, 2013; Walter et al., 2015; Gracia-Romero et al., 2019). Depending on the type of sensor and how the information gathered is used, measurements can be related to different morphological and physiological traits relevant to the phenotypic performance of the crop. Currently, the implementation of low-cost conventional cameras to formulate vegetation indices (VIs) derived from red–green–blue (RGB) images (i.e., information from the visible range) is increasingly successful for studying aspects related to canopy green biomass (Fernandez-Gallego et al., 2019b). Sensors measuring spectral information at the red and near-infrared (NIR) region are also commonly used to evaluate plant biomass and greenness, as the normalized difference vegetation index (NDVI) (Rouse et al., 1976), whereas pigment content and photosynthetic capacity can be assessed by the measurement of more specific bands at the visible region, as the photosynthetic reflectance index (PRI) (Tambussi et al., 2002). Plant water status is also assessed by using specific bands, in this case within the NIR region, informing on leaf turgidity, as the water band index (Penuelas et al., 1993). Canopy temperature (CT) is another phenotypic trait to consider when assessing crop water availability, because CT informs on crop transpiration and water use (Jackson et al., 1988; González-Dugo et al., 2006). Nevertheless, phenotyping is not necessarily restricted to the use of an array of different remote sensing techniques, as several analytical (i.e., laboratory) traits may also be very useful. For example, the analysis of stable carbon (δ^13^C) isotope composition, when performed on plant dry matter informs about the water regimen of the wheat crop (Farquhar and Richards, 1984; Araus et al., 2003b).

At present, one of the major challenges for successful implementation of HTPP to define ideotypes lies in unlocking the potential of the huge amounts of data generated by high-throughput phenotyping platforms (Coppens et al., 2017). Machine learning (ML) aims to interpret data by the development of algorithms built from training sets (van Klompenburg et al., 2020), and these are being used increasingly in agricultural applications. Indeed, ML applications may also be helpful for the simultaneous integration of miscellaneous phenotypic data (Hall et al., 2022). Recent literature highlights the opportunities found in combining data from different technologies to assist HTPP (Fiorani and Schurr, 2013; Granier and Vile, 2014; Costa et al., 2019; Araus et al., 2022).

The novelty of this study, with respect to the recently published works (e.g. Senapati and Semenov, 2019; Ullah et al., 2020; Olivoto and Nardino, 2021), is that it defines, for durum wheat, which ideotypic characteristics, assessed through different remote sensing approaches, contribute to crop adaptation over a wide range of Mediterranean conditions. Thus, the ideotypes were defined in terms of the best combination of remote sensing traits predicting yield and assessed at anthesis and grain filling, two critical phenological moments in terms of water and heat stress occurrence, for wheat under Mediterranean conditions. The remote sensing traits informed on crop water status, photosynthetic efficiency, and growth/stay green. A set of current (i.e., post–green revolution) durum wheat cultivars widely grown in Spain during the last four decades was evaluated for three consecutive crop seasons across a wide range of latitudes with very diverse climatic conditions and in trials under different growing conditions (normal planting under support irrigation and rainfed conditions and late planting under support irrigation). The contribution of each parameter into random forest regression models built separately for each environment and phenological stage was used to select the best set of remote sensing traits determining GY. On the basis of those results, environment-specific GY-predicting models were defined; furthermore, we evaluated whether the addition of the carbon isotope composition of mature grains improved the prediction accuracies of these models.

## 2 Materials and methods

### 2.1 Experimental design and varieties

**Figure 1:**
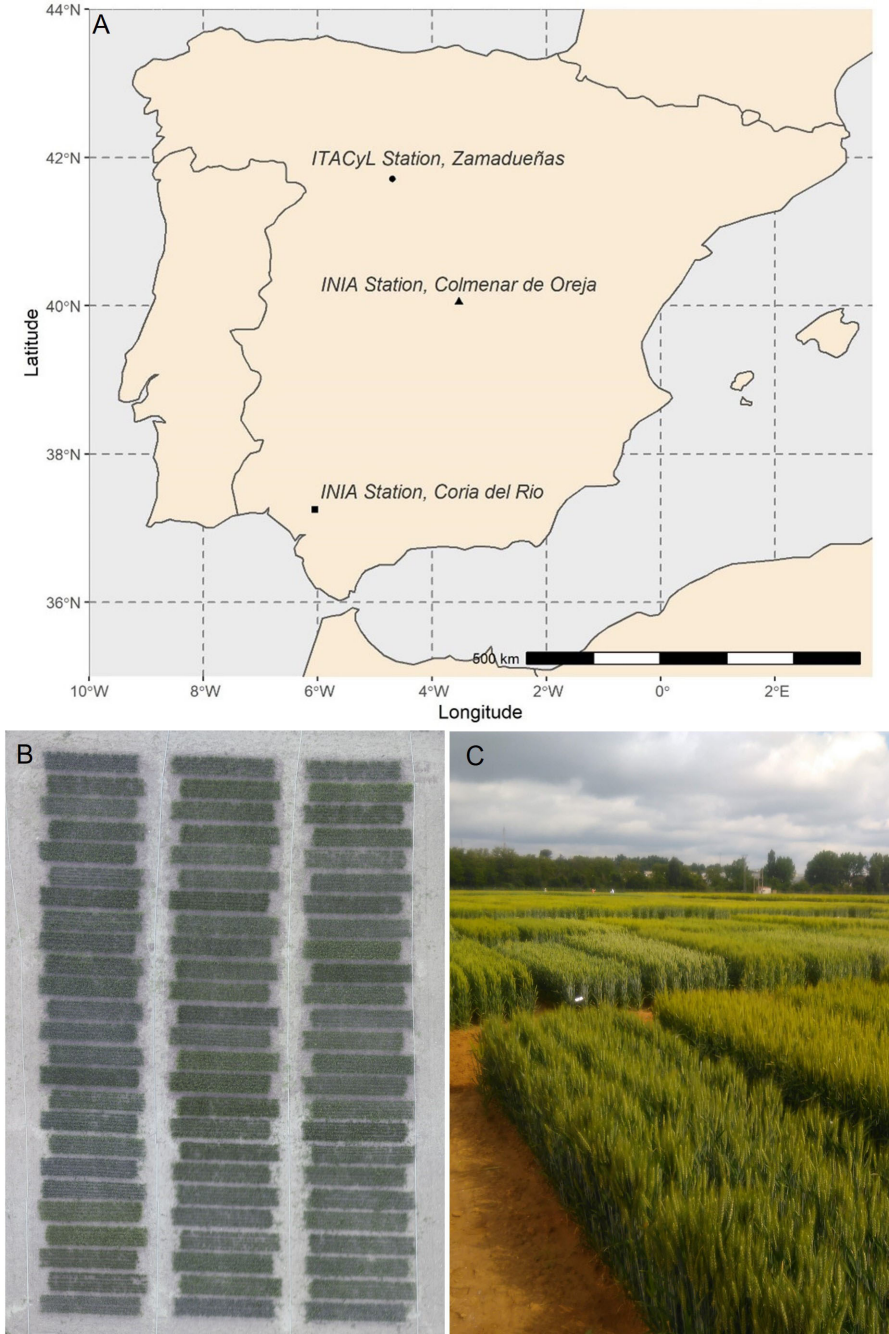
**(A)** Map of Spain with the locations of the experimental stations. **(B)** Example of an aerial view of one of the trials, corresponding to the late planting conditions of Aranjuez in 2018. **(C)** Panoramic view from the ground of the field trials in Valladolid, image taken in May of 2018.

Experiments were carried out under field conditions in three experimental stations located across a wide range of latitudes in Spain (**Figure 1A**): two belonging to the Spanish “Instituto Nacional de Investigación y Tecnología Agraria y Alimentaria” (INIA) and located in Coria del Rio, Seville (37°14′N, 06°03′W, 5 masl), and Colmenar de Oreja–Aranjuez, Madrid [40°04′N, 3°31′W, 590 meters above the sea level (masl)], and one at the headquarters of the “Instituto Tecnológico Agrario de Castilla y León” (ITACyL) in Zamadueñas, Valladolid (41°41′N, 04°42′W, 700 masl) during three consecutive crop seasons between 2016 and 2019. The plant material consisted in a panel of 24 semi-dwarf varieties of durum wheat [*Triticum turgidum L*. subsp *durum* (Desf) Husn.] marketed in Spain during the last four decades (**Supplemental Table 1**). Trials were established in a complete block design with three replicates (**Figure 1B**) and plot consisted of seven rows planted 20 cm apart and a seed rate of 250 seeds m^−2^, representing an area of 7 m × 1.4 m (**Figure 1C**).

Climatic data from the different crop seasons at each experimental station were recorded through the Spanish platform SIAR (Servicio de Informacion Agroclimática para el Regadío; www.siar.es) from meteorological stations next to the fields. Monthly temperature and rainfall averages are plotted in **Supplemental Figure 1**. The experiment sites covered a wide range of Spanish latitudes, and, thus, the climatic conditions were very diverse during the different crop seasons. The combination of different locations, agronomic conditions, and years were considered as 19 different environments (**Table 1**) to assess the performance of the chosen genotypes. Such differences were represented by daily mean temperatures and water inputs (precipitation and irrigation). In the case of Coria, the crop cycles were characterized by high temperatures during the crop cycle (average temperatures 14.5°C, 13.1°C, and 14.4°C, during the three consecutive seasons) and a wide range of accumulated precipitation during the crop season from 349.6 mm (2016/2017), 595.4 mm (2017/2018), to 146.6 mm (2017/2018), and an accumulated evapotranspiration of around 486.7, 478.5, and 554.2 mm, respectively, for each crop season. The environmental conditions of the trials in Aranjuez were rather constant in terms of temperature, with lower values than in Coria (11.4°C, 9.5°C, and 10.4°C, for the three consecutive seasons, respectively), and, despite the site being located in a semiarid environment, the annual variability in precipitation was high (107.9 mm, 321.1 mm, and 108.2 mm for the three consecutive years), whereas the potential evapotranspiration values were around 611.7 mm, 553.4 mm, and 788.0 mm. Finally, Valladolid experienced temperatures comparable to Aranjuez (11.7°C, 9.6°C, and 10.1°C) alongside a strong annual variation in precipitation during the crop cycle (107.3 mm, 402.5 mm, and 103.3 mm, for the three consecutive seasons, respectively) and a potential evapotranspiration of around 593.0 mm, 479.5 mm, and 555.5 mm, respectively. Data soil analysis is included in **Supplemental Table 2**.

**Table 1:** Agronomic information for each study site during each growing season.

L	T	Y	Sowing date	Harvest date	Irr.	Prec.	Total water	Basic dressing	Top dressing
					(mm)	(mm)	(mm)	(8-15-15 NPK kg ha^−1^)	(46% urea kg ha^−1^)
Coria del Rio	**Rainfed**	**16/17**	14/12/2016	12/06/2017	0	349.6	349.6	450_(12/12/2016)_	227_(15/03/2017)_
		**17/18**	20/12/2017	19/06/2018	0	595.4	595.4	450_(18/12/2017)_	227_(13/03/2018)_
		**18/19**	18/12/2019	18/06/2019	0	146.6	146.6	450_(14/12/2018)_	227_(14/03/2019)_
Aranjuez	**Irrigation**	**16/17**	14/12/2016	19/07/2017	395	107.97	502.97	450_(16/12/2016)_	227_(15/03/2017)_
		**17/18**	28/11/2017	04/07/2018	140	321.13	461.13	450_(23/11/2017)_	185_(28/02/2019)_
		**18/19**	29/11/2018	28/06/2019	540	108.2	648.2	450_(23/11/2017)_	230_(27/02/2018)_
	**Rainfed**	**16/17**	14/12/2016	19/07/2017	0	107.97	107.97	450_(16/12/2016)_	227_(15/03/2017)_
		**17/18**	28/11/2017	04/07/2018	0	321.13	321.13	450_(26/02/2018)_	185_(28/02/2019)_
		**18/19**	29/11/2018	28/06/2019	0	108.2	108.2	450_(26/11/2018)_	230_(27/02/2018)_
	**Late**	**16/17**	01/03/2017	19/07/2017	425	51.49	476.19	450_(16/12/2016)_	227_(15/03/2017)_
		**17/18**	26/02/2018	10/07/2018	220	228.31	448.31	450_(26/11/2018)_	185_(16/04/2019)_
		**18/19**	27/02/2019	05/07/2019	680	79.4	759.4	450_(23/02/2019)_	230_(23/04/2018)_
Valladolid	**Irrigation**	**16/17**	29/11/2016	06/07/2017	155	107.3	262.3	300_(07/11/2016)_	150_(17/02/2017)_ + 150_(21/03/2017)_
		**17/18**	13/11/2017	25/07/2018	110	420.5	420.5	300_(12/11/2017)_	150_(20/02/2018)_ + 150_(17/04/2018)_
		**18/19**	03/12/2018	15/07/2019	153	103.3	103.3	300_(16/11/2018)_	150_(28/02/2019)_ + 150_(12/04/2019)_
	**Rainfed**	**16/17**	29/11/2016	06/07/2017	55	107.3	162.3	300_(07/11/2016)_	150_(17/02/2017)_ + 150_(21/03/2017)_
		**17/18**	23/11/2017	20/07/2018	0	420.5	420.5	300_(12/11/2017)_	150_(20/02/2018)_ + 150_(17/04/2018)_
		**18/19**	03/12/2018	03/07/2019	0	103.3	103.3	300_(16/11/2018)_	150_(28/02/2019)_ + 150_(12/04/2019)_
	**Late**	**16/17**	09/02/2017	20/07/2017	155	67.3	222.38	300_(07/11/2016)_	150_(21/03/2017)_

L, location; T, trial; Y, year. The letters "NPK" stand for nitrogen, phosphorus, and potassium. NPK, nitrogen, phosphorus, and potassium.

### 2.2 Data collection

For each environment, remote sensing measurements were performed at two sampling dates corresponding to the phenological stages of anthesis and grain filling (**Table 2**). Days after sowing (DAS) together with growing degree days (GDD) were counted until each sampling. GDD was calculated as follows:

**Table 2:** Agronomic information for each study site during each growing season. L, location; T, trial; Y, year; DAS, days after sowing; GDD, growing degree days.

L	T	Y	Sampling date	Phenological Stage	DAS	GDD
Coria del Rio	**Rainfed**	**2016/2017**	05/04/2017	Anthesis	112	1360.12
			25/04/2017	Grain filling	132	1680.20
		**2017/2018**	18/04/2018	Anthesis	119	1440.45
			15/05/2018	Grain filling	146	1884.88
		**2018/2019**	04/04/2019	Anthesis	107	1425.84
			02/05/2019	Grain filling	135	1863.46
Aranjuez	**Irrigation**	**2016/2017**	04/05/2017	Anthesis	133	2399.68
			18/05/2017	Grain filling	147	2767.24
		**2017/2018**	16/05/2018	Anthesis	169	1387.76
			28/05/2018	Grain filling	181	1622.30
		**2018/2019**	13/05/2019	Anthesis	165	1511.91
			27/05/2019	Grain filling	179	1769.29
	**Rainfed**	**2016/2017**	04/05/2017	Anthesis	133	2399.68
			18/05/2017	Grain filling	147	2767.24
		**2017/2018**	16/05/2018	Anthesis	169	1387.76
			28/05/2018	Grain filling	181	1622.30
		**2018/2019**	13/05/2019	Anthesis	165	1511.91
			27/05/2019	Grain filling	179	1769.29
	**Late**	**2016/2017**	18/05/2017	Anthesis	78	1813.76
			06/06/2017	Grain filling	97	2423.69
		**2017/2018**	28/05/2018	Anthesis	91	1622.30
			11/06/2018	Grain filling	105	1864.20
		**2018/2019**	27/05/2019	Anthesis	89	1769.29
			11/06/2019	Grain filling	104	2070.86
Valladolid	**Irrigation**	**2016/2017**	16/05/2017	Anthesis	168	1382.38
			07/06/2017	Grain filling	190	1794.34
		**2017/2018**	17/05/2018	Anthesis	185	1176.57
			13/06/2018	Grain filling	212	1599.35
		**2018/2019**	15/05/2019	Anthesis	163	1274.48
			29/05/2019	Grain filling	177	1473.95
	**Rainfed**	**2016/2017**	16/05/2017	Anthesis	168	1382.38
			07/06/2017	Grain filling	190	1794.34
		**2017/2018**	17/05/2018	Anthesis	534	1176.57
			13/06/2018	Grain filling	212	1599.35
		**2018/2019**	15/05/2019	Anthesis	163	1274.48
			29/05/2019	Grain filling	177	1473.95
	**Late**	**2016/2017**	16/05/2017	Anthesis	96	1382.38
			07/06/2017	Grain filling	118	1794.34


$$GDD=\sum^{}\left(\left(\frac{T_{max}-T_{min}}{2}\right)-T_{base}\right)$$

where T_max_ and T_min_ corresponds to the highest and the lowest daily temperature, respectively, and the T_base_ used was 0°C.

The set of sensors and cameras used, the VIs measured (**Table 3**), and the laboratory analyses are described in the following sections. VIs have been categorized into three distinct groups on the basis of the specific trait they measure: (1) indices related to plot greenness and biomass, (2) indices related to leaf/canopy pigment content and photosynthesis, and (3) indices related to water status.

**Table 3:** Sensors and cameras used during this experiment and their major specifications, with the traits assessed and the indices used with their formulations.

Trait	Sensor	Major specifications	Indices	Formula	References
Canopy greenness and biomass	RGB camera - Sony ILCE-QX1 (Sony Corporation, Minato, Japan)	16 Megapixels; sensor size: 17.3 mm × 13.0 mm; focal length: 35 mm. Trigged and exposure time programed in automatic mode.	a*	–	(Pointer, 2009)
			Green area (GA)	60°< Hue< 180°	(Casadesús et al., 2007)
	Handheld multispectral sensor GreenSeeker crop sensor (Trimble, Sunnyvale, CA, USA)	Wavelength range: 670 nm and 840 nm; field of view: 25 cm (1 m from the canopy).	Normalized difference vegetation index (NDVI)	$\frac{R_{780}-R_{670}}{R_{780}+R_{670}}$	(Rouse et al., 1976)
Leaf pigment content	Leaf-clip sensor: Dualex (Force-A, Orsay, France)	Measured area: 5 mm in diameter; sample thickness: 1 mm maximum; light sources: 5 LED; 1 UV-A, 1 red, and 2 NIR	Chlorophylls a + b (Chl)	$\frac{NIR\;trans.\;-\;Red\;trans.}{Red\;trans.}$	(Cerovic et al., 2012)
			Flavonoids (Flav)	$\log\frac{NIR\;fluor.\;excited\;red}{NIR\;fluor.\;excited\;UV-A}$	
			Anthocyanin (Anth)	$\log\frac{NIR\;fluor.\;excited\;red}{NIR\;fluor.\;excited\;green}$	
			Nitrogen balance index (NBI)	$\frac{Chl}{Flav}$	
Photosynthetic capacity	Multispectral camera - Tetracam micro-MCA12 (Tetracam Inc., Chats-worth, CA, USA)	Incident light sensor (ILS): 15.6 megapixels; wavelengh range: 450 nm to 950 nm; sensor size: 6.66 mm × 5.32 mm. Wavelength	Transformed chlorophyll absorption index (TCARI)	$0.3\cdot \left(R_{700}-R_{670}\right)-0.2\cdot \left(R_{700}-R_{550}\right)\cdot \left(\frac{R_{780}}{R_{670}}\right)$	(Haboudane et al., 2002)
			TCARI/OSAVI ratio	$\frac{TCARI}{OSAVI*}$	
			Anthocyanin reflectance index (ARI2)	$R_{840}\cdot \left(\frac{1}{R_{550}}-\frac{1}{R_{700}}\right)$	(Gitelson et al., 2001)
			Carotenoid reflectance index (CRI2)	$\left(\frac{1}{R_{550}}-\frac{1}{R_{700}}\right)$	(Gitelson et al., 2002)
			Photochemical reflectance index (PRI)**	$\frac{R_{550}-R_{570}}{R_{550}+R_{570}}$	(Gamon et al., 1992)
			Chlorophyll carotenoid index (CCI)	$\frac{R_{550}-R_{670}}{R_{550}+R_{670}}$	(Gamon et al., 2016)
			Water band index (WBI)	$\frac{R_{970}}{R_{900}}$	(Penuelas et al., 1993)
Water status	FLIR Tau2 640 thermal imaging camera	With a VOx uncooled microbolometer equipped with a TeAx Thermal Capture 2.0; temperature range: −55°C to 95°C; wavelength range: 7.5 µm to 13.5 µm.	Canopy temperature (CT)	–	(Costa et al., 2013)

UV-A, ultraviolet A; NIR, near-infrared; R, reflectance; trans., transmittance; fluor., fluorescence; OSAVI, optimized soil adjusted vegetation index.

*The formula of the OSAVI index used in the TCARI/OSAVI ratio is the following: $\frac{R_{780}-R_{670}}{R_{780}+R_{670}+0.16}$ . **PRI, R_550_ is used instead of the original R_531_. LED. light-emitting diode.

GY (Mg ha^−1^) was determined for the entire plot using a harvester.

### 2.3 Leaf pigments

The content of different leaf pigments was assessed at ground level using a Dualex sensor (Force-A, Orsay, France), which operates with a red reference beam at 650 nm and a UV light at 375 nm (Cerovic et al., 2012). This sensor produces measurements of chlorophylls a + b (Chl), flavonoids (Flav), and anthocyanin (Anth) content and also calculates the nitrogen balance index (NBI), which is the ratio of Chl/Flav related to the nitrogen and carbon allocation. For each data of measurement and plot, five different measurements on the flag leaf from the main stem on five different flag leaves from the main stem of five different plants were performed. The measurements were taken from the middle portion of the leaf lamina.

### 2.4 RGB images and derived vegetation indices

**Figure 2:**
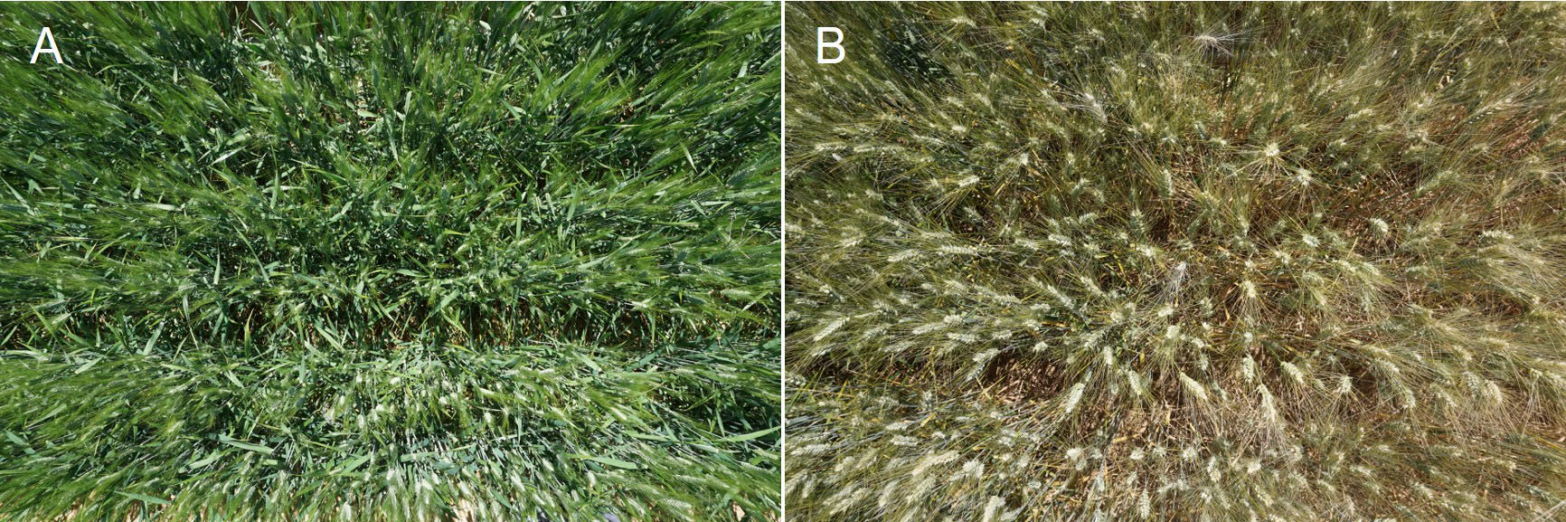
Examples of RGB images of wheat plots acquired from the ground corresponding to the irrigated conditions of Valladolid in 2019 at the grain filling and late grain filling stages. Image **(A)** was taken during the anthesis stage and image **(B)** during the grain filling stage.

VIs derived from RGB images were evaluated for each plot using a 20.1-megapixel Sony ILCE-QX1 camera (Sony Corporation, Minato, Japan) attached to a Sony Monopod VCTMP1 (Sony Corporation, Minato, Japan). The distance to the crop canopy was adjusted to 1 m (**Figure 2**). Color calibration of both cameras with ColorChecker Passport Photo (X-Rite, Inc., USA) reported determination coefficients (R^2^) between 0.88 and 0.94 for all the RGB parameters (data not shown). Processing of RGB images for calculation of the VIs in relation to different color properties of potential interest was performed with MosaicTool (https://www.gitlab.com/sckefauver/MosaicTool; University of Barcelona, Barcelona, Spain) integrated as a plugin for FIJI (Fiji is Just ImageJ; https://www.fiji.sc/Fiji/) (Gracia-Romero et al., 2019). From the HSI (hue–saturation–intensity) color space, the portion of pixels classified as green by their Hue values (referring to the color tint) was determined by the green area (GA) index (Casadesús et al., 2007). The GA corresponds to the percentage of pixels that have a hue value between 60° and 180°. From the CIELab color space models [recommended by the International Commission on Illumination (CIE) for improved color chromaticity compared to HSI color space], the parameter a* was calculated, which represents the red–green spectrum of chromaticity (Pointer, 2009).

### 2.5 Multispectral vegetation indices

Ground-based multispectral sensing was conducted through measurements with a GreenSeeker crop sensor (Trimble, Sunnyvale, CA, USA), by passing it over the middle of each plot at a constant height of 0.5 m above and perpendicular to the canopy to calculate the NDVI. In addition, aerial assessments were performed using a Tetracam micro-MCA (multiple camera array) 12 (Tetracam Inc., Chatsworth, CA, USA), which consists of 12 independent image sensors and optics, each with user configurable filters (450 nm ± 40 nm, 550 nm ± 10 nm, 570 nm ± 10 nm, 670 nm ± 10 nm, 700 nm ± 10 nm, 720 nm ± 10 nm, 780 nm ± 10 nm, 840 nm ± 10 nm, 860 nm ± 10 nm, 900 nm ± 20 nm, and 950 nm ± 40 nm). The 12th sensor is a dedicated ILS (incident light sensor) that faces upward and uses microfilters to provide an accurate band-by-band reflectance calibration in real-time. PixelWrench II version 1.2.2.2 (Tetracam, Chatsworth, CA, USA) was used to pre-process the multi-spectral images by aligning and calibrating each band. A suite of multispectral indices was calculated from the different bands using custom code developed in FIJI and integrated within the MosaicTool software (**Table 3**).

**Figure 3:**
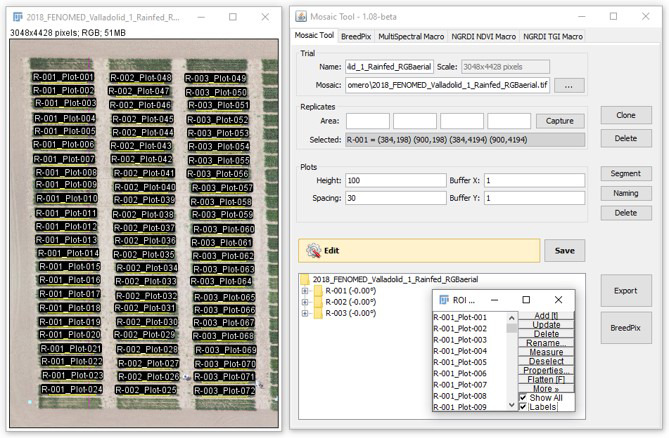
For the extraction and processing of information at the single plot level, we used the Mosaic Tool (University of Barcelona Plugin for FIJI), which is a software package for semi-automatic image segmentation of aerial images for UAV plant phenotyping studies (http://www.sckefauver.com/software-development/ and http://www.sckefauver.com/software-development/ and http://www.integrativecropecophysiolo-gy.com/software-development/).

The flights were performed using a 6S12 XL oktokopter (HiSystems GmbH, Moomerland, Germany) under clear sky conditions, with image data captured at an altitude of 50 m. The unmanned aerial vehicle (UAV) have an active two-servo gimbal that was used to correct for the effect of pitch and roll movements during the flight. Pre-processed aerial images from each sensor were combined to obtain an accurate orthomosaic by producing a three-dimensional reconstruction with Agisoft PhotoScan Professional software (Agisoft LLC, St. Petersburg, Russia; http://www.agisoft.com/) (Bendig et al., 2014). To that end, images with at least 80% overlap were used. Then, regions of interest corresponding to each plot were segmented and exported using the MosaicTool (**Figure 3**).

### 2.6 Stable carbon and nitrogen isotope composition and total C and N contents

Mature grains collected at harvest were dried at 60°C for a minimum of 48 h and pulverized to a fine powder, from which 1 mg was enclosed in tin capsules and analyzed using an elemental analyzer (Flash 1112 EA; ThermoFinnigan, Schwerte, Germany) coupled with an isotope ratio mass spectrometer (Delta C IRMS, ThermoFinnigan), operating in continuous flow mode at the Scientific and Technical facilities of the University of Barcelona (Centres Científics i Tecnológics de la Universitat de Barcelo-na, CCiTUB). The ^13^C/^12^C ratios of plant material were expressed in δ notation as stable carbon isotope composition (δ^13^C) as follows:


$$\delta^{13}C=\left[\left(\frac{R_{sample}}{R_{standard}}\right)-1\right]*1,000$$

where R_sample_ refers to plant material and R_standard_ refers to Pee Dee Belemnite calcium carbonate. International isotope secondary standards of a known ^13^C/^12^C ratio (IAEA CH7, polyethylene foil, IAEA CH6 sucrose, and USGS 40 l-glutamic acid) were calibrated against Vienna Pee Dee Belemnite calcium carbonate with an analytical precision of 0.1‰.

### 2.7 Statistical analysis

Statistical analysis was performed using the open-source software R and RStudio 1.0.44 (R Foundation for Statistical Computing, Vienna, Austria). Means and standard errors of the agronomic data were calculated from the three replicates of each experimental condition (year × location × treatment × genotype). The effects of treatment conditions, location, growing seasons, genotypes, and their interaction with GY and the remote sensing measurements were determined through a four-factor analysis of variance (ANOVA). Differences were considered significant at p-values ≤0.05. Pearson correlation coefficients were used to analyze the relationship between the remote sensing parameters and GY.

GY-predicting models were developed for each environment, first, by combining all the parameters measured separately at anthesis and at grain filling using random forest regression. Random forest regression is a ML that works by creating multiple decision trees on randomly sampled subsets of the data and then combining the results of these trees to make a final prediction. In our study, we performed random forest regression using the random forest package, with 500 decision trees and five variables used for splitting at each node. The model was trained on a randomly selected 70% of the data and validated on the remaining 30%. We evaluated the performance of the model using R-squared. In addition, we performed feature importance analysis to determine the relative importance of each variable in predicting the outcome. The described workflow was repeated 100 times, and the final R-squared and importance values were defined as the mean of these measurements across all 100 runs. Then, on the basis of the coefficient of correlation and the importance features from the random forest analysis, the top three parameters at each phenological stage were selected and were combined to identify the best parameters combination using only anthesis, grain filling, or both stages. Finally, isotope composition was added to the best models to check whether this improved the prediction accuracy.

## 3 Results

### 3.1 Genotype × environment interactive effect on GY

The combined analysis of variance across years, locations, management treatments, and genotypes revealed that mean squares were significant for GY (**Table 4**). Considering all the experiments between the 2016/2017 to 2018/2019 crop seasons, most of the variance was caused by the management conditions of the trials, followed by the factors of year and location. The genotype factor accounted for a low but significant effect on GY.

**Table 4:** Analysis of variance for grain yield (GY) based on the set of cultivars across the locations, management trials, and crop seasons assessed.

Source of variation	Mean Square	P-value	%CTV
Year (Y)	506.34	***	28.62
Location (L)	262.85	***	14.86
Trial (T)	635.17	***	35.90
Genotype (G)	4.29	***	0.24
L × T	9.24	***	0.52
L × G	2.09	***	0.12
T × G	1.17	***	0.07
L × T × G	1.16	***	0.07
L × Y × G	183.95	***	10.40
L × Y × T	1.35	***	0.08
L × Y	87.72	***	4.96
Y × T	70.3	***	3.97
Y × G	1.47	***	0.08
Y × T × G	0.68	ns	0.04
L × Y × T × G	0.73	ns	0.04
Residuals	0.61		0.03

Values presented are the mean square values, the p-values, and the calculation of the percentage contribution to total variation (CTV). Significance levels: ns, not significant; ***P< 0.001. Y, year; L, location; T, trial; G, genotype.

The ANOVA comparison between all genotypes for each experimental trial is presented at **Table 5** and the rankings of the highest- and lowest-yielding genotypes at **Supplemental Table 3**. Significant genotypic differences in GY were reported in all trials, except for the irrigation and rainfed conditions of Valladolid in 2016/2017, the rainfed conditions of Aranjuez in 2017/2018, and the rainfed conditions of Valladolid in 2017/2018. The irrigation trials were the best-yielding environments in each location, achieving the highest yields in Valladolid during the 2018/2019 (Olivadur, 9.06 ± 0.66 Mg ha^−1^ as the top genotype) and 2019/2020 (Avispa, 9.02 ± 0.30 Mg ha^−1^ as the top genotype) crop seasons. The next highest-yielding conditions were in Coria during 2016/2017 (Don Ricardo, 8.52 ± 0.51 Mg ha^−1^ as the top genotype) and, after that, the irrigation trial in Aranjuez during 2017/2018 (Mexa, 8.28 ± 0.38 Mg ha^−1^ as the top genotype). In contrast, the lowest-yielding trials were those grown under rainfed conditions. In particular, the lowest yields were achieved in the rainfed trials in Aranjuez during 2018/2019 (Simeto, 0.80 ± 0.06 Mg ha^−1^ as the top genotype). To a lesser extent than the rainfed environments, the late-planting trials also contributed to yield reductions relative to the well-irrigated trials at the same locations.

**Table 5:** ANOVA analysis of the effect of the genotypes tested on grain yield (GY) and its heritability (H^2^) across the growing seasons.

Location	Treatment	Crop season	GY p-value	GY H^2^
Coria	Rainfed	2016/2017	***	0.876
		2017/2018	***	
		2018/2019	*	
Aranjuez	Irrigation	2016/2017	**	0.729
		2017/2018	**	
		2018/2019	***	
	Rainfed	2016/2017	**	0.813
		2017/2018	ns	
		2018/2019	**	
	Late planting	2016/2017	***	0.57
		2017/2018	***	
		2018/2019	***	
Valladolid	Irrigation	2016/2017	ns	0.257
		2017/2018	*	
		2018/2019	***	
	Rainfed	2016/2017	ns	0.834
		2017/2018	ns	
		2018/2019	ns	
	Late planting	2016/2017	***	–

ns, no significant, p > 0.05; *p < 0.05; **p < 0.01; ***p < 0.001.

### 3.2 Individual and combined remote sensing indices for GY prediction

**Figure 4:**
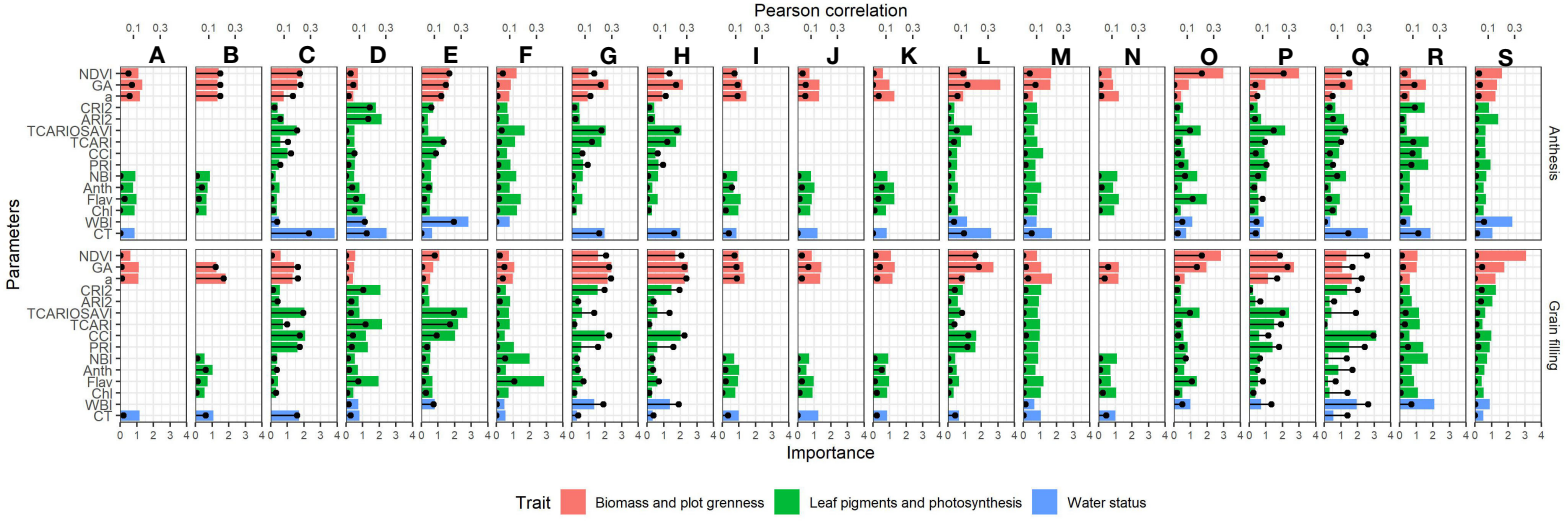
Bar graph showing the average importance weights of the remote sensing parameters used in the rainforest prediction models. Values are means from the 100 runs developed for each model. The y-axis displays the parameters used in the model, whereas the x-axis at the bottom represents their importance. The top x-axis shows the coefficient of determination of the parameters against yield, and it is indicated with the black lines and dots. Environments are ordered from the lowest (upper left) to the highest (lower right) average yield as follows: **(A)** 2019-Aranjuez-Rainfed, **(B)** 2019-Valladolid-Rainfed, **(C)** 2017-Aranjuez-Rainfed, **(D)** 2017-Valladolid-Rainfed, **(E)** 2018-Aranjuez-Rainfed, **(F)** 2018-Aranjuez-Late, **(G)** 2017-Aranjuez-Late, **(H)** 2017-Valladolid-Late, **(I)** 2019-Coria-Rainfed, **(J)** 2019-Aranjuez-Late, **(K)** 2019-Aranjuez-Irrigation, **(L)** 2017-Aranjuez-Irrigation, **(M)** 2018-Coria-Rainfed, **(N)** 2019-Valladolid-Irrigation, **(O)** 2018-Valladolid-Irrigation, **(P)** 2018-Valladolid-Rainfed, **(Q)** 2017-Valladolid-Irrigation, **(R)** 2018-Aranjuez-Irrigation, and **(S)** 2017-Coria-Rainfed. Orange bars correspond to measures related to biomass and canopy greenness, green bars correspond to measures of leaf-pigments and photosynthetic capacity/efficiency, and blue bars correspond to measures of water status. Acronyms of the parameters are defined in **Table 3**.

The performance of the different remote sensing indices predicting GY varied significantly across environments and depending on the phenological moment assessed (**Figure 4**). The remote sensing indices included in this study were grouped in three categories attending the physiological process (crop water status, flag leaf and canopy photosynthetic efficiency/capacity, and crop growth/greenness) putatively related with final yield. Canopy greenness indicators were reported as the best traits in the model predicting GY for most of the environments and regardless of the phenological moment assessed. Thus, NDVI, a*, and GA reported very similar correlations against GY (with averaged values of R^2 = ^0.20 at anthesis and R^2 = ^0.21 during grain filling, in average). The contribution of the canopy greenness indices was consistent also across the growing treatments assessed, and high correlations against GY were reported for irrigated treatments (as for the 2017 grain filling evaluations of Aranjuez with GA: R^2 = ^0.46 and Valladolid with NDVI: R^2 = ^0.64), rainfed treatments (as for the 2017 anthesis evaluations of Aranjuez with GA: R^2 = ^0.45 and for the 2018 grain filling evaluations of Valladolid with GA: R^2 = ^0.58), and late planting (as for the 2017 grain filling evaluations of Aranjuez with GA: R^2 = ^0.56 and Valladolid with NDVI:R^2 = ^0.56). Regarding flag leaf pigments and photosynthetic efficiency/capacity traits, correlations in low-yielding environments were reported for both anthesis and grain filling, whereas, for higher-yielding environments, the correlations were higher at grain filling. Within this trait category, the best correlations were found for multispectral indices informing on canopy photosynthetic capacity, with the most repeated parameter being the TCARI/OSAVI (transformed chlorophyll absorption index/optimized soil adjusted vegetation index) (R^2 = ^0.45 and R^2 = ^0.48 for the irrigation conditions from Valladolid in 2017 at anthesis and grain filling, respectively; and R^2 = ^0.49 for the rainfed conditions from Aranjuez in both 2017 and 2018 during grain filling). Moreover, indices informing on the photosynthetic efficiency at the canopy level also performed well as GY predictors in some environments. This was the case for the multispectral indices PRI and, to a greater extent, CCI. Those indices reported moderate-to-good correlations against GY especially under environments with higher temperatures and water stress and relatively high temperatures as the rainfed conditions of Aranjuez in 2017 (R^2 = ^0.30 at anthesis and R^2 = ^0.44 at grain filling) or the heat conditions of the late-planting cultivars from Valladolid in 2017 R^2 = ^0.16 at anthesis and R^2 = ^0.56 at grain filling). In the case of the flag leaf pigment readings from the Dualex (informing on photosynthetic leaf performance), the correlations against GY were only present at the grain filling evaluations of the normal planting irrigation conditions from Valladolid in 2017 (Chl: R^2 = ^0.35; Anth: R^2 = ^0.42; and NBI: R^2 = ^0.34). Finally, for the water status evaluations, CT was highly correlated against GY specially at anthesis under the lowest-yielding environments (as in 2017 for the rainfed conditions of Aranjuez, R^2 = ^0.58), whereas the multispectral index WBI (water band index) (also informing on crop water status) correlated better at grain filling for the environments with medium (as in 2017 for the late-planting conditions of Aranjuez, R^2 = ^0.48) to higher (as in 2017 for the irrigation conditions of Valladolid, R^2 = ^0.66) yielding potential.

**Figure 5:**
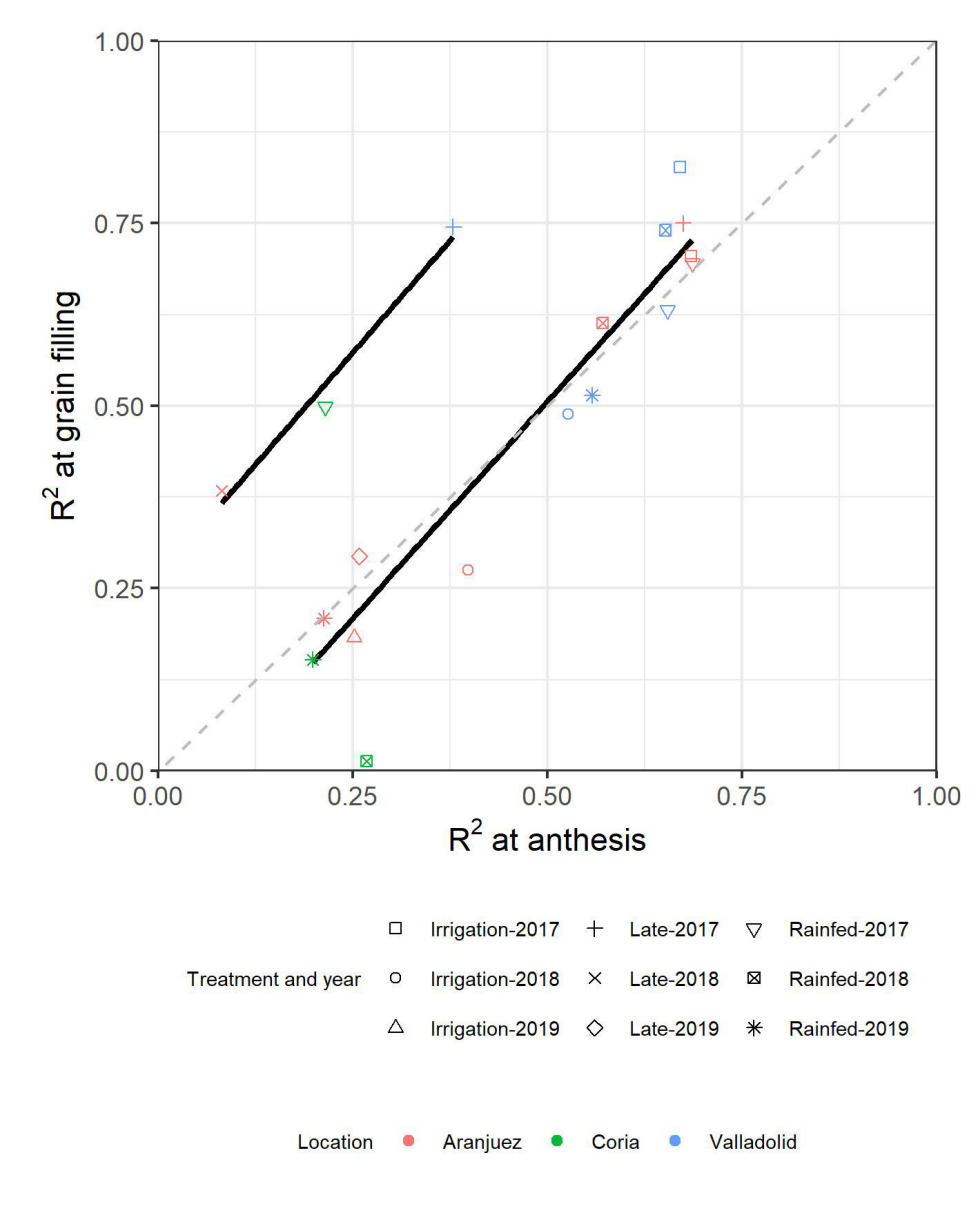
Relationships across environments between the determination coefficients (R^2^) from GY-predicting models developed at anthesis and the R^2^ from the models developed at grain filling.

For a better understanding of the relevance of each of the parameters measured in each environment at each of the phenological moments, rainforest regression models were developed combining all the parameters measured. For most of the environments, the strength of the models was very similar for both anthesis and grain filling (**Figure 5**). Late-planting conditions from Valladolid in 2017 and from Aranjuez in 2018 together with the trial from Coria in 2017 followed a different trend, as prediction at grain filling was reported to be more accurate than at anthesis. The predictions of Coria in 2018 were also displaced from the general trend, as the prediction failed for the grain filling evaluations. The model with the highest accuracy was achieved under the irrigated conditions of Valladolid in 2017 at grain filling (R^2 = ^0.87). Moreover, the low accuracies reported in 2019 matched the models with less predictors, as the multispectral indices [NDVI, TCARI, TCARI/OSAVI, ARI2 (Anthocyanin reflectance index), CRI2 (carotenoid reflectance index), PRI (photochemical reflectance index), CCI (chlorophyll carotenoid index), and WBI] were missing for those campaigns.

**Figure 6:**
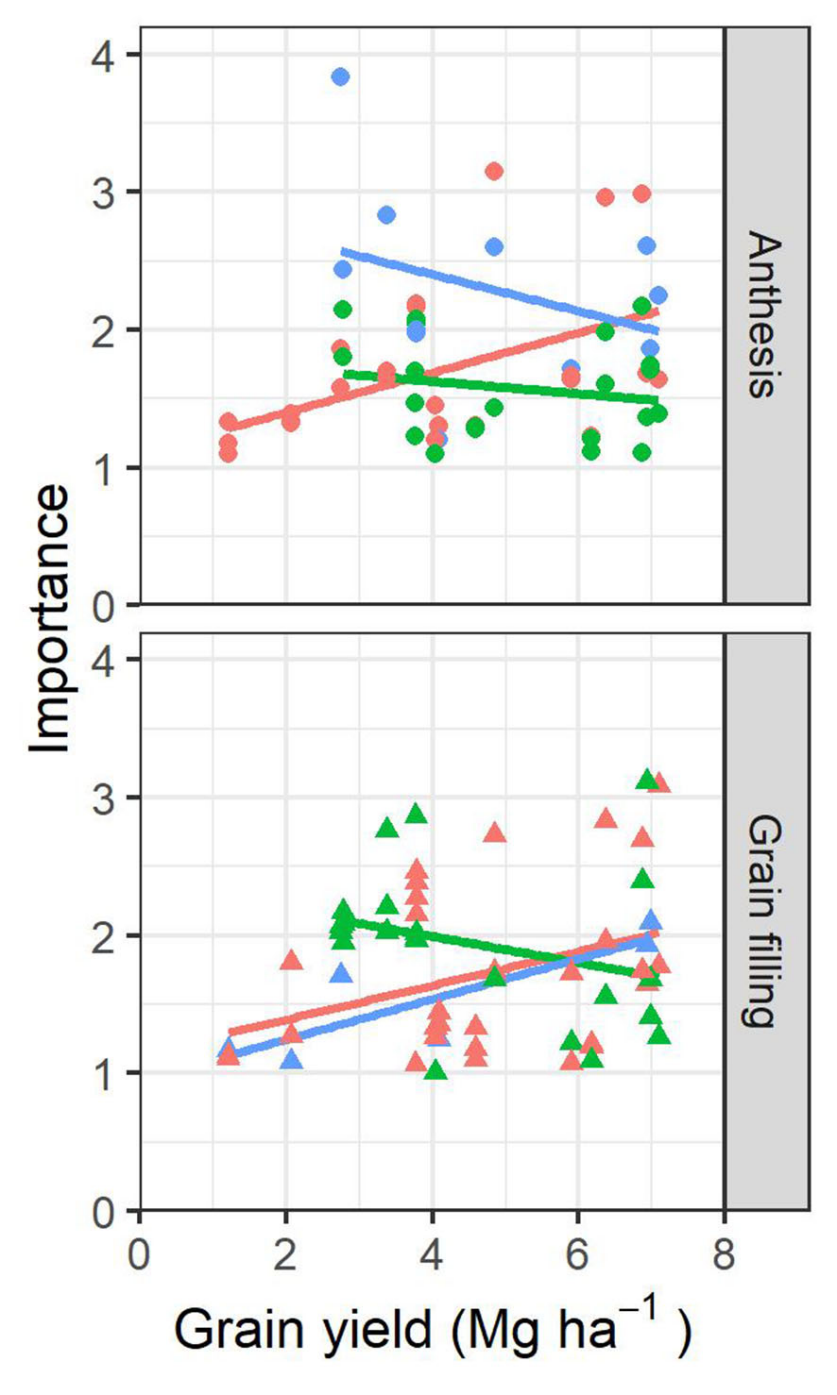
Scatterplot showing the relationship between the top three parameters with the highest importance weight from each environment and the average grain yield (GY) of each environment. The color of the points and fitting lines corresponds to the kind of trait assessed: orange corresponds to measures related to biomass and canopy greenness, green corresponds to measures of leaf-pigments and canopy photosynthetic efficiency/capacity, and blue corresponds to measures of water status.

Using a normalized relative importance metric obtained by the random forest regression model, the relative contribution of each variable used in the prediction with the presence of all other variables in the model was evaluated. Roughly, the importance of each parameter within the prediction model followed a very similar trend as to how well the parameter alone was correlated with the yield. When variables were ranked according to their importance, the most repeated variables identified were GA-measured at grain filling (9/19 environments), a* (6/19 environments), and GA (5/19 environments) measured at anthesis and CT-evaluated at anthesis (6/19 environments). **Figure 6** represents the tendencies of how the weight in the models from the most important parameters for each environment evolve as the average GY for the environment increases. Biomass and plot greenness parameters clearly increased their relevance in the models for both anthesis and grain filling stages as the environments are more productive, whereas the trends of the leaf pigment content and photosynthetic parameters showed a reduction. The water status parameters were less important during anthesis as the average GY from the environments increased, while, during grain filling, they were more important.

### 3.3 Proposed GY-predicting models as the guidelines for ideotype definition

To study the synergies of the traits describing the highest-yielding cultivars for each of the environments assessed, all possible combinations of the top three parameters based on their importance in the rainforest models at each phenological stage were used to describe GY regression models. The generated models were ordered according to the Bayesian information criterion for selecting the best predictive model considering both the goodness of the fit of the model and the complexity of the models (**Supplemental Table 4**; one category of models only used anthesis data, other category only used grain filling data, and another combined both. For most cases, models incorporating measures at both anthesis and grain filling improved the prediction accuracies when compared with using just a single phenological moment. The highest accuracies for high-yielding environments were reported for Valladolid for the irrigation conditions in 2017 (*GA_anthesis_*, a^*^_grain filling_, *CCI_grain filling_*; R^2 = ^0.76), for the irrigation conditions in 2018 (*Flav_anthesis_*, *NDVI_anthesis_*, *NDVI_grain filling_*; R^2 = ^0.55), and for the rainfed conditions in 2019 (*NDVI_anthesis_*, a^*^_grain filling_; R^2 = ^0.46). On the other hand, for the lowest-yielding environments, the highest accuracies were reported for Valladolid for the rainfed conditions in 2018 (*PRI_anthesis_*, *GA_grain filling_*, *PRI_grain filling_*; R^2 = ^0.66), for Aranjuez for the rainfed conditions in 2017 (*CT_anthesis_*, *NDVI_anthesis_*; R^2 = ^0.64), and for the late-planting conditions in 2017 (*GA_grain filling_*, *PRI_grain filling_*; R^2 = ^0.62).

**Figure 7:**
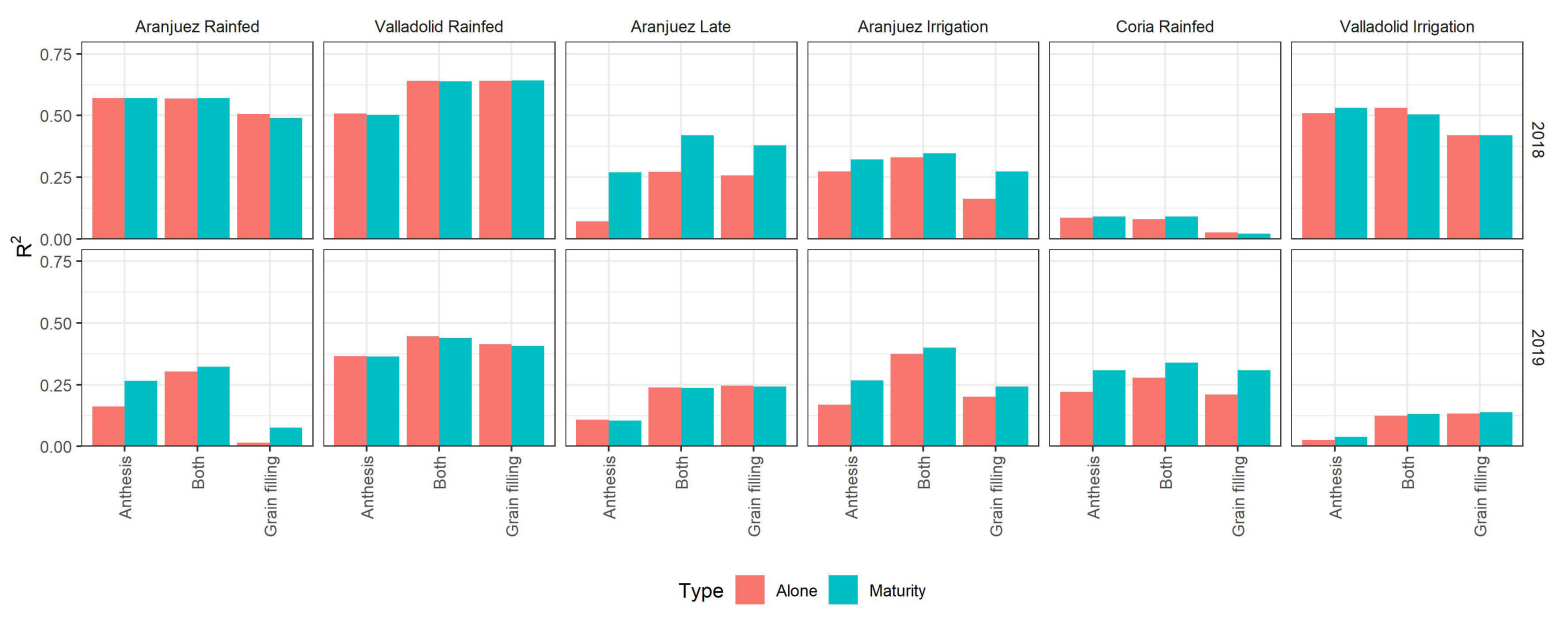
Bar graph comparison of the accuracies of the best prediction-models with and without the addition of the carbon stable isotope composition of mature kernels. The graph displays the Pearson correlation coefficient for the models using data from anthesis, grain filling, and data from both stages. Orange bars correspond to the models using the remote sensing measures alone, and the blue bars correspond to the same models but with the addition of the isotope data. The combinations of location and growing conditions are ordered from the lowest to highest yields.

We further tested for the 2017/2018 and 2018/2019 crop seasons if the accuracies of the best models predicting GY improved by including the stable carbon isotope composition of mature kernels (**Figure 7**). The determination coefficients of the Pearson correlations between the carbon isotope composition and GY are presented in **Supplemental Table 5**. Overall, a very slight improvement was reported especially for the environments with lower prediction accuracies when only remote sensing parameters were used. Best improvements were achieved under the late-planting conditions of Aranjuez 2018 and the trial of Coria 2019. However, for environments with already accurate prediction models but with lower yields, as the rainfed conditions of Aranjuez and Valladolid in 2018, the addition of δ^13^C did not improve the predictions.

## 4 Discussion

The aim of the present study was to determine the ideotypic guidelines for plant phenotyping of well-adapted genotypes to different Mediterranean environments, based on the analysis of a set of remote sensing traits measured during the reproductive stage. For that, a total of 19 of environments exhibiting a wide range of growing conditions and several fold differences in GY were tested. The strategy pursued was to identify which combination of specific traits is the most critical for developing GY-predicting models for each environmental growing condition. Results proved how the complexity of G × E effects on the genotypes performance hampered the correct selection of the most accurate phenotypic traits to define specific environment ideotypes, reporting significant differences in the genotypic performance in terms of the relation between GY and the remote sensing traits assessed. In addition to that, the study also supports the rather low genotypic variability existing among the post–green revolution durum wheat cultivars grown in the Euro Mediterranean region, which makes the objective of defining ideotypes and phenotyping protocols more challenging.

### 4.1 Environment effect on genotypic performance of GY and traits

Under Mediterranean conditions, water regime and temperature have been described as factors explaining the major portion of GY variation in cereals (Voltas et al., 2002; Senapati and Semenov, 2020; de Lima et al., 2021). The drought stress experienced by the rainfed trials greatly reduced GY in comparison with the irrigated trials at the same locations. In fact, year-to-year variation in weather had a large impact on the GY of rainfed trials (except for the trial at Coria, which it is benefitted from its proximity to the Guadalquivir River), as the quantity and distribution of rainfall during the three cropping seasons were markedly different. Climatic variability in precipitation and temperature is known to affect crop yields. A delay in the planting date implies higher temperatures during the entire crop cycle and particularly during the reproductive and grain filling phases of wheat (Farooq et al., 2011). This reduces the duration of the crop cycle and increases respiration rates and eventually the occurrence of heat stress, which overall decreases the GY relative to normal planting dates supported by irrigation conditions. Because the average GY under water or temperature stress conditions was reduced in comparison with the normal planting support irrigation conditions, the importance of breeding for resilience to these stresses is emphasized (Juliana et al., 2019). In this sense, genotypic variability in GY was highly significant but relatively minor compared to the effect of growing conditions. This is in spite of the fact that the set of 24 cultivars tested comprehensively covered the genetic advance in durum wheat achieved in Spain during the last four decades (Chairi et al., 2018; Chairi et al., 2020a; Chairi et al., 2020b). The rather small range of genotypic variability in GY of durum wheat contrasts with the far larger variability for bread wheat (Acreche et al., 2008), and this is probably related to the difference in ploidy between the two species (Mastrangelo and Cattivelli, 2021). Nevertheless and in spite of the rather narrow range of genotypic variability, the models performed overall well in predicting GY, which highlights the strength of the approach.

### 4.2 Under which circumstances are the measures of green biomass and delayed senescence relevant to predict cultivars performance?

In the case of an abiotic stress such as water stress, the first evidence of its negative effect on GY was reported by the VIs because this stress limits leaf expansion and then crop growth and canopy photosynthesis. As a measure of crop growth, aboveground green biomass assessed through RGB VIs such as GA and a* and the multispectral NDVI has been proposed as a useful selection trait for GY improvement of wheat under Mediterranean conditions (Fernandez-Gallego et al., 2019b). In the current work, both NDVI and RGB VIs assessed at anthesis generated quantitative assessments of canopy cover that indicated a high contribution to the GY-predicting models. NDVI combines low reflectance in the visible region of the spectrum (400–700 nm) and high reflectance in the NIR (700–1,100 nm) region (Hassan et al., 2019), effectively assessing vegetative cover and vigor. Furthermore, the calculation of RGB indices based on the color properties of the canopy as the GA and the a* was also reported as strong predictors of GY across most of the environments assessed regardless of their yield potential, which agrees with previous studies evaluating GY under Mediterranean conditions through color indicator VIs (Fernandez-Gallego et al., 2019b; Gracia-Romero et al., 2019). Moreover, when measured during grain filling, such VIs monitor the duration of leaf/canopy photosynthesis and become a critical way of detecting cultivars with delayed senescence (Lopes and Reynolds, 2012; Christopher et al., 2014; Gracia-Romero et al., 2019; Anderegg et al., 2020), especially when measured as a response to water stress. Canopy greenness evaluations were one of the most important traits across the environments, particularly when measured during grain filling, which, therefore, informs on stay-green. Delaying senescence and maintaining canopy greenness have been reported as being positively correlated to the final GY (Gregersen et al., 2013). However, only functional stay-green is of interest for crop improvement, meaning that photosynthesis and accumulation of assimilates in harvestable tissues (i.e., grains) should be prolonged (Christopher et al., 2016). However, under environments without major growth limitations, which corresponded to the years with higher precipitation at Coria or the irrigated trials at Aranjuez and Valladolid, the importance of those indicators of the aboveground biomass during the grain filling phases was reduced. Probably, the saturation pattern of NDVI and even that of the RGB VIs (Fernandez-Gallego et al., 2019b; Gracia-Romero et al., 2023) may reduce the accuracy of the prediction under conditions where green biomass is high.

### 4.3 What is the role of water status traits in defining cultivar performance?

Two remote sensing parameters were used to assess water status at canopy level. One is the turgor hydration of the leaves thought the multispectral index WBI (Blank et al., 2021) and the other one the plant transpiration activity, *via* the CT measurement (Araus et al., 2002). For most of the environments and regardless of their yield potential, both parameters were repetitively selected among the most critical parameters for GY predictions. However, there are some substantial differences between those measures that may respond to the results reported and may help to better apply them. First, CT provides an instantaneous proxy of crop water conditions, and any stress that induces stomatal closure will be translated into a decrease in transpiration and a consequent increase in CT (Araus et al., 2003a). Significant negative correlations of CT measurements with GY were reported for all the environments studied, suggesting that even trials characterized by high yields (e.g., the support irrigation trials at normal planting dates or the trials at Coria) exhibited some degree of water stress. CT measurements have been widely reported as an effective tool to assess genotypic responses to stress and, thus, are a good predictor of yield (Fischer et al., 1998). Meanwhile, multispectral indices like the WBI provide a more integrated measure of stress over a longer period of time and eventually more severe levels of water stress (Das et al., 2021) that already led to a reduction of water and of cell turgor in the leaves causing reflectance changes in specific regions of the NIR. Thus, CT may be more sensitive, to fast responses to water stress, involving stomatal closure, whereas WBI revealed loss of turgor and changes in hydration level of the leaf and, therefore, longer-term and/or more severe water stress. Therefore, both remote sensing indicators together may cover different levels of water stress. Another proxy used as an integrative indicator of cultivar water status during the crop cycle is the carbon isotope composition (δ^13^C) analyzed in mature kernels (Araus et al., 2003a; Araus et al., 2013). In fact, δ^13^C was negatively correlated with GY for most of the environments, whether a low, mild, or high stress was imposed, supporting δ^13^C as a powerful selection tool for Mediterranean conditions (Araus et al., 2003a, Araus et al., 2013). However, the addition of δ^13^C to the prediction models based on remote sensing did not further improve the performance for most of the prediction models. Therefore, these remote sensing–based prediction models are already covering most of the variability in GY accounted by water conditions.

### 4.4 Are indices informing on leaf pigment and canopy photosynthetic capacity/efficiency valid by themselves to predict phenotypic performance?

The relevance of traits informing on leaf pigment content and crop photosynthetic efficiency at the canopy level in predicting GY under most of the environments assessed was proven in the results. In contrast to the indices classified as estimators of greenness and biomass (GA and NDVI), this category includes indices that measure leaf pigments based on absorbance measurements and multispectral indices at the canopy level, which use more bands in the visible part of the spectrum. This difference makes these indices more sensitive to variations in pigment content and photosynthetic capacity beyond just plot greenness and biomass, which is the primary focus of the NDVI. However, those indices were unusually reported as the most important or the highest correlated traits in the models, whereas the most relevant traits predicting yield were connected to crop biomass, greenness, or water status. Among the individual leaf traits, chlorophyll content of the flag leaf was one of the traits more times present in the GY-predicting models. This can be interpreted as Chl readings offer to the prediction models a complementary information for the identification of high-yielding cultivars beyond differences in canopy coverage/greenness, as leaf chlorophyll content has been frequently reported as a good indicator of the senescence-induced response (Neufeld et al., 2006; Xiong et al., 2015). A comparable situation was observed with the multispectral indices related to the photosynthetic capacity/efficiency, such as PRI, CCI, CRI2, or ARI2. When evaluated at canopy level, these indices were selected repeatedly in the GY-predicting models, but usually after traits informing on crop biomass/greenness and water status. In fact and in addition to informing on photosynthetic efficiency, these indices when assessed at the canopy level are highly influenced by the amount of green biomass. This was evidenced by the appearance of those indices on some of the models together with biomass indicators like in 2017-Valladolid-Irrigation (*GA_anthesis_*, a^*^_grain filling_, *CCI_grain filling_*; R^2 = ^0.75) or together with water status measures like 2017-Aranjuez-Rainfed (*CT_anthesis_*, *CCI_grain filling_*; R^2 = ^0.62). Those indices work using narrow bands related to pigment absorption (Garbulsky et al., 2011), providing a valuable addition to the potential canopy photosynthesis derived from the vegetation density and greenness. PRI and CCI are related to photosynthesis efficiency, being the PRI more sensitive to changes in the amount of light absorbed by pigments such as chlorophyll and carotenoids (Garbulsky et al., 2011), whereas CCI is more used for estimations of chlorophyll (Gamon et al., 2016). Whereas ARI2 and CRI2 indices inform about the amount of anthocyanins and carotenoids, respectively, both pigments with photoprotection roles (Gitelson et al., 2001; Gitelson et al., 2002; Santini et al., 2019).

Despite this general tendency, exceptions were found in some of the most stressed (and lowest yielding) environments, where parameters related to leaf pigments and canopy photosynthetic capacity/efficiency were chosen by the models before the other remote sensing parameters. Under severe rainfed conditions that markedly reduced yield (2017-Aranjuez-Rainfed, 2017-Valladolid-Rainfed, and 2018-Aranjuez-Rainfed), estimations during grain filling of multispectral indices measuring photosynthetic capacity (PRI and CCI) and pigment content (TCARI/OSAVI and TCARI) at the canopy level outperformed any other parameters. In addition, the contribution of Flav assessed at leaf level was relevant in some of the low-yielding environments (2018-Aranjuez-Late), as an increase in flavonoids content can be associated to a protective response indicating that cultivars with a greater capacity to withstand stress exhibited higher contents of these protective pigments prior to senescence (Ma et al., 2014a).

### 4.5 Formulation of ideotype recommendations for each agro-environment based on measurement performance

In environments with mild to moderate water limitations and with moderate temperatures at anthesis (provided by normal planting and support irrigation conditions), most productive genotypes reported higher canopy green biomass as indicated by the VIs at both anthesis and grain filling. During the reproductive stages, genotypes may also reach a point where the differences in vegetation greenness are negligible because the indices are saturated by dense canopies (Duan et al., 2017). For that matter, and in agreement with previous studies (Wu et al., 2010; Magney et al., 2016), proposed ideotypes must be also screened using a higher photosynthetic efficiency parameters, as it was done in this study through multispectral indices as PRI and CCI. However, canopy greenness may become important again later during grain filling because indices indicating constitutive stay green attitude and thus longer grain filling periods (Christopher et al., 2016; Fernandez-Gallego et al., 2019b) are associated with higher-yielding genotypes.

Under growing conditions characterized by elevated temperatures during the crop cycle, even if under irrigation (as in the case of late planting), wheat cultivars reporting larger and/or greener canopies, as assessed by higher NDVI or GA, and higher photosynthetic efficiency around anthesis (higher PRI and CCI) were the most productive. Moreover, the content of either leaf flavonoids or anthocyanins was also present in the prediction models for late planting. Although the literature has variable observations on the accumulation of photoprotection pigments in response to stress (Chakraborty and Pradhan, 2012; Hammad and Ali, 2014), increases in flavonoids or anthocyanins are expected to induce tolerance to moderate heat stress and then help maintain yield (Ma et al., 2014b). In the case of Coria trials, even if planted at a normal date, represented a warmer scenario in than the other two locations. For conditions of elevated temperatures during the grain filling, represented by both late planting and Coria, the delay of senescence, assessed by the greener VIs was the main trait defining yield, indicating the stay-green ability as mitigation strategy for the harmful aspects of terminal heat (Latif et al., 2020). However, in general, the prediction models performed poorer for the environments with short cycles (i.e., late planting, or Coria) compared with the normal planting, suggesting the speed of the phenological development represented a limitation for an efficient assessment of the remote sensing traits.

Finally, when growing conditions cause severe yield losses due to water stress, the measurement of actual canopy greenness and stay green behavior will be not enough to define the best performing genotypes. In addition to a higher crop green biomass across the crop cycle (higher NDVI or GA), a higher transpiration and water use at anthesis (i.e., lower CT) is also incorporated to the prediction models. Under conditions with low and erratic rainfall without any irrigation, the definition of drought-tolerant wheat ideotypes relies on the capability of genotypes to maintain stomatal aperture (Sinclair et al., 2017; Chenu et al., 2018). This agrees with the concept that effective use of water is a key factor associated with higher productivity under Mediterranean environments (Blum, 2009). Moreover, models were also benefited by VIs estimating pigments related to stress tolerance (Ahmed et al., 2019).

## 5 Conclusions

Combining different remote sensing traits based on the targeted environmental (climate and management) conditions may improve HTPP. In this study, the assessment of different physiological traits *via* remote sensing approaches plus subsequent precise selection of more critical traits *via* ML served not only to develop predictive models but also to delineate what physiological traits define ideotypes of durum wheat across a wide range of Mediterranean conditions varying in water availability and temperatures. Against this background, common traits critical to GY under environments with mild to moderate limiting constraints included higher index values for crop cover and canopy greenness throughout the reproductive stage as well as indicators of a better water status. Under severe stress conditions found under rainfed conditions, in addition to the key attributes already mentioned, the contribution in the prediction models of indices informing on photosynthetic capacity/efficiency and photoprotection pigments increased, clearly complementing the information of traits informing on biomass and water status. However, if the stress is only generated by high temperatures, then the delay of senescence was the major trait defining GY.

Overall, regardless of the growing conditions, high-yielding wheat cultivars reported similar behavior in (i) reaching higher biomass during anthesis, (ii) further maintaining green biomass during grain filling (higher VIs values indicating stay green behavior) and (iii) better water status and higher water use in terms of higher stomatal conductance and transpiration (lower CT and δ^13^C), and (iv) the translation of these factors to higher crop yield.

Further advances in HTTP will come from a more plastic strategy for phenotyping, combining for each target environment, specific remote sensing indices (informing on vegetation cover, water status, or pigment content) measured at a given phenological stage. Such approach may deliver a comprehensive understanding of the cultivar’s adaptation to specific environments.

## Data availability statement

The raw data supporting the conclusions of this article will be made available by the authors, without undue reservation.

## Author contributions

AG-R: Conceptualization, Formal Analysis, Investigation, Methodology, Visualization, Writing – original draft, Writing – review & editing. TV: Data curation, Formal Analysis, Methodology, Writing – review & editing. SK: Investigation, Methodology, Software, Supervision, Writing – review & editing. FR: Formal Analysis, Investigation, Methodology, Writing – review & editing. JS: Investigation, Methodology, Writing – review & editing. MN-T: Investigation, Writing – review & editing. NA: Investigation, Writing – review & editing. JA: Conceptualization, Funding acquisition, Investigation, Writing – review & editing, Writing – original draft.
