# Supplementary material for: Defining durum wheat ideotypes adapted to Mediterranean environments through remote sensing traits
Source: Front Plant Sci. 2023 Sep 5;14:1254301. doi: 10.3389/fpls.2023.1254301 (PMC10508639; doi:10.3389/fpls.2023.1254301)
Supplement: Supplementary file 1 [file DataSheet_1.docx]

Supplementary Material

# Supplementary Tables

**Supplemental Table 1**. Set of modern semi-dwarf durum wheat cultivars tested in this study with year of release, country of origin and available information on provenance and/or pedigree.

| **Genotype** | **Year of release** | **Country** | | **Provenance/Pedigree** | |
| --- | --- | --- | --- | --- | --- |
|  |  |  | |  | |
| Mexa | 1980 | Spain | | GERARDO-VZ-469/3/JORI(SIB)//ND-61-130/LEEDS | |
| Vitron | 1983 | Spain | | TURCHIA-77/3/JORI-69(SIB)/(SIB)ANHINGA//(SIB)FLAMINGO | |
| Simeto | 1988 | Italy | | CAPEITI-8/VALNOVA[1620][1622][1623][1625][1666] | |
| Gallareta | 1994 | Spain | | RUFF/FLAMINGO//MEXICALI-75/3/SHEARWATER | |
| Pedroso | 1993 | Spain | | Batlle seeds | |
| Regallo | 1990 | Spain | | Diputación General de Aragón | |
| Arcobaleno | 1996 | Spain | | Chen/Altar84 | |
| Claudio | 1998 | Italy | | SEL.CIMMYT-35/DURANGO//ISEA-1938/GRAZIA | |
| Burgos | 1999 | Spain | | SUDDEUTSCHE SAATZ | |
| Dorondon | 1990 | Spain | | Genética y Gestión,S.C. | |
| Avispa | 2001 | Italy | | Limagrain-CIMMYT | |
| Amilcar | 2002 | Spain | | ZEGZAG-1/LUNDE-5//GREENSHANK-32 | |
| Saragolla | 2004 | Italy | | Iride/0114 | |
| Solea | 2005 | Spain | | Monsanto Agriculture Spain | |
| Euroduro | 2007 | Spain | | IRTA | |
| Don Ricardo | 2008 | Spain | | Agrovegetal-CIMMYT | |
| Core | 2009 | Spain | | Eurogen, PROSEME seeds | |
| Kiko Nick | 2009 | Spain | | SEL.CIMMYT-35/DURANGO//ISEA-1938/GRAZIA | |
| Sculptur | 2011 | France | | RAGT Semence | |
| Athoris | 2011 | Italy | | Limagrain Europe | |
| Don Norman | 2012 | Spain | | Agrovegetal - CIMMYT | |
| Olivadur | 2013 | Spain | | RAGT 2N SAS seeds | |
| Iberus | 2014 | Spain | | Agromonegros | |
| Haristide | 2015 | France | | Caussade Semences S.A. | |
|  |  | |  | |  |

**Supplemental table 2**. Soil information for each study site.

|  | **Depth (cm)** | **pH** | **Type of soil** | **Organic matter** | | **CaCO_3_ (%)** | **Coarse sand (%)** | **Fine sand (%)** | **Silt (%)** | **Clay (%)** |
| --- | --- | --- | --- | --- | --- | --- | --- | --- | --- | --- |
|  |  |  |  | **C (%)** | **N (%)** |  |  |  |  |  |
| *Valladolid* | 10 | 8.5 | Loam | 1.35 | 0.065 | < 3 |  | 34 | 48 | 17.5 |
| *Aranjuez* | 10 | 8.4 | Clay-loam | 0.58 | 0.1 | 13.7 | 3.1 | 11 | 51 | 38 |
| *Coria* | 20 | 7.8 | Silty clay loan | 1.96 | 0.17 | 24.57 | 0.5 | 10.5 | 55 | 30 |

**Supplemental table 3**. Average grain yield (Mg ha-1) of the top three highest yielding genotypes and the top 3 lowest yielding genotypes for each of the 19 environments tested.

| **L** | **T** | **Y** | **Highest yielding genotype** | | | | **Lowest yielding genotype** | | | |
| --- | --- | --- | --- | --- | --- | --- | --- | --- | --- | --- |
| **Coria** | **Rainfed** | **2016/2017** | *DRicardo* | 8.52 | ± | 0.51 | *Simeto* | 5.83 | ± | 0.24 |
|  |  |  | *Amilcar* | 8.26 | ± | 0.04 | *Core* | 5.07 | ± | 0.26 |
|  |  |  | *Euroduro* | 8.22 | ± | 0.32 | *Pedroso* | 4.74 | ± | 0.26 |
|  |  | **2017/2018** | *Olivadur* | 6.65 | ± | 0.05 | *Pedroso* | 4.16 | ± | 0.05 |
|  |  |  | *KikoNick* | 6.46 | ± | 0.28 | *Gallareta* | 4.58 | ± | 0.38 |
|  |  |  | *Athoris* | 6.46 | ± | 0.25 | *Vitron* | 4.95 | ± | 0.03 |
|  |  | **2018/2019** | *Athoris* | 5.08 | ± | 0.07 | *Arcobaleno* | 3.41 | ± | 0.59 |
|  |  |  | *Iberus* | 4.80 | ± | 0.33 | *Saragolla* | 3.37 | ± | 0.43 |
|  |  |  | *KikoNick* | 4.73 | ± | 0.39 | *Pedroso* | 3.08 | ± | 0.25 |
| **Aranjuez** | **Irrigation** | **2016/2017** | *Olivadur* | 6.03 | ± | 0.42 | *DNorman* | 4.10 | ± | 0.22 |
|  |  |  | *Burgos* | 5.67 | ± | 0.53 | *Arcobaleno* | 4.05 | ± | 0.26 |
|  |  |  | *Sculptur* | 5.34 | ± | 0.08 | *Core* | 3.46 | ± | 0.10 |
|  |  | **2017/2018** | *Mexa* | 8.28 | ± | 0.38 | *Pedroso* | 5.93 | ± | 0.68 |
|  |  |  | *Amilcar* | 8.25 | ± | 0.29 | *Olivadur* | 5.51 | ± | 0.78 |
|  |  |  | *Vitron* | 7.97 | ± | 0.21 | *Haristide* | 3.83 | ± | 1.17 |
|  |  | **2018/2019** | *Amilcar* | 5.75 | ± | 0.22 | *Haristide* | 3.52 | ± | 0.43 |
|  |  |  | *Dorondon* | 5.38 | ± | 0.71 | *Sculpdur* | 3.50 | ± | 0.28 |
|  |  |  | *Euroduro* | 5.34 | ± | 0.31 | *Pedroso* | 3.48 | ± | 0.18 |
|  | **Rainfed** | **2016/2017** | *Olivadur* | 3.58 | ± | 0.30 | *Saragolla* | 2.27 | ± | 0.32 |
|  |  |  | *Athoris* | 3.28 | ± | 0.20 | *Solea* | 1.82 | ± | 0.14 |
|  |  |  | *Claudio* | 3.22 | ± | 0.24 | *Core* | 1.61 | ± | 0.20 |
|  |  | **2017/2018** | *KikoNick* | 4.19 | ± | 0.15 | *Solea* | 2.97 | ± | 0.43 |
|  |  |  | *Avispa* | 3.93 | ± | 0.43 | *Saragolla* | 2.65 | ± | 0.53 |
|  |  |  | *Claudio* | 3.91 | ± | 0.14 | *Haristide* | 2.49 | ± | 0.38 |
|  |  | **2018/2019** | *Solea* | 1.65 | ± | 0.07 | *Regallo* | 0.87 | ± | 0.06 |
|  |  |  | *Olivadur* | 1.63 | ± | 0.05 | *Pedroso* | 0.87 | ± | 0.22 |
|  |  |  | *Amilcar* | 1.62 | ± | 0.09 | *Simeto* | 0.80 | ± | 0.06 |
|  | **Late** | **2016/2017** | *Euroduro* | 5.06 | ± | 0.16 | *Pedroso* | 3.06 | ± | 0.17 |
|  |  |  | *Burgos* | 4.87 | ± | 0.27 | *Simeto* | 3.05 | ± | 0.42 |
|  |  |  | *Claudio* | 4.62 | ± | 0.19 | *Core* | 2.95 | ± | 0.19 |
|  |  | **2017/2018** | *Core* | 4.98 | ± | 0.12 | *Sculpdur* | 2.76 | ± | 0.24 |
|  |  |  | *KikoNick* | 4.75 | ± | 0.22 | *Haristide* | 2.57 | ± | 0.38 |
|  |  |  | *Athoris* | 4.63 | ± | 0.26 | *Olivadur* | 2.27 | ± | 0.16 |
|  |  | **2018/2019** | *Euroduro* | 5.65 | ± | 0.36 | *Haristide* | 3.34 | ± | 0.27 |
|  |  |  | *Solea* | 5.02 | ± | 0.14 | *Pedroso* | 3.14 | ± | 0.26 |
|  |  |  | *KikoNick* | 4.62 | ± | 0.36 | *Simeto* | 2.99 | ± | 0.12 |
| **Valladolid** | **Irrigation** | **2016/2017** | *Arcobaleno* | 7.81 | ± | 0.05 | *Amilcar* | 6.23 | ± | 0.59 |
|  |  |  | *Olivadur* | 7.81 | ± | 0.53 | *Core* | 6.23 | ± | 0.64 |
|  |  |  | *Mexa* | 7.53 | ± | 0.53 | *Pedroso* | 5.92 | ± | 0.25 |
|  |  | **2017/2018** | *Haristide* | 7.82 | ± | 0.52 | *Saragolla* | 5.77 | ± | 0.25 |
|  |  |  | *Olivadur* | 7.20 | ± | 0.30 | *Pedroso* | 5.74 | ± | 0.52 |
|  |  |  | *Claudio* | 6.94 | ± | 0.13 | *Simeto* | 5.50 | ± | 0.15 |
|  |  | **2018/2019** | *Olivadur* | 9.06 | ± | 0.66 | *DRicardo* | 5.01 | ± | 1.39 |
|  |  |  | *Athorix* | 8.07 | ± | 0.77 | *Vitron* | 4.91 | ± | 0.44 |
|  |  |  | *Avispa* | 7.88 | ± | 0.92 | *Sculptur* | 4.21 | ± | 0.26 |
|  | **Rainfed** | **2016/2017** | *Arcobaleno* | 3.79 | ± | 0.66 | *Euroduro* | 1.94 | ± | 0.33 |
|  |  |  | *Dorondon* | 3.66 | ± | 0.28 | *Pedroso* | 1.75 | ± | 1.40 |
|  |  |  | *Claudio* | 3.57 | ± | 0.08 | *Iberus* | 1.71 | ± | 0.70 |
|  |  | **2017/2018** | *Amilcar* | 8.41 | ± | 0.19 | *Pedroso* | 6.03 | ± | 0.31 |
|  |  |  | *Burgos* | 7.86 | ± | 0.68 | *Simeto* | 5.85 | ± | 0.81 |
|  |  |  | *Sculpdur* | 7.85 | ± | 0.59 | *Saragolla* | 5.23 | ± | 0.85 |
|  |  | **2018/2019** | *DRicardo* | 2.90 | ± | 0.48 | *Haristide* | 1.46 | ± | 0.16 |
|  |  |  | *Arcobaleno* | 2.73 | ± | 0.07 | *Euroduro* | 1.42 | ± | 0.12 |
|  |  |  | *Solea* | 2.52 | ± | 0.46 | *Olivadur* | 1.40 | ± | 0.21 |
|  | **Late** | **2016/2017** | *Athoris* | 6.93 | ± | 0.59 | *Gallareta* | 4.12 | ± | 0.76 |
|  |  |  | *Olivadur* | 6.54 | ± | 0.41 | *Pedroso* | 4.09 | ± | 0.32 |
|  |  |  | *Euroduro* | 6.19 | ± | 0.36 | *Simeto* | 4.09 | ± | 0.55 |

**Supplemental table 4**. Multiple regression models to predict grain yield (GY) using data from anthesis alone, grain filling alone and combining both phenological models. R^2^, coefficient of determination; RSME, root square mean error; Bayesian information criterion (BIC).

| L | T | Y | Model | R^2^ | RMSE | BIC | p.value | Phenological stage |
| --- | --- | --- | --- | --- | --- | --- | --- | --- |
| Aranjuez | Irrigation | 2017 | CCI_1_GA_2_NDVI_2_GY_2 | 0.52 | 0.52 | 122.05 | 0.000 | Both |
|  |  |  | GA_2_GY_2 | 0.46 | 0.55 | 124.29 | 0.000 | Grain filling |
|  |  |  | GA_1_NDVI_1_GY_2 | 0.32 | 0.62 | 142.99 | 0.000 | Anthesis |
|  |  | 2018 | CT_1_PRI_2_GY_2 | 0.33 | 1.04 | 223.42 | 0.000 | Both |
|  |  |  | CT_1_GY_2 | 0.27 | 1.08 | 225.99 | 0.000 | Anthesis |
|  |  |  | WBI_2_GY_2 | 0.16 | 1.16 | 236.22 | 0.000 | Grain filling |
|  |  | 2019 | Flav_1_a_1_a_2_GY_2 | 0.37 | 0.65 | 158.71 | 0.000 | Both |
|  |  |  | Anth_1_a_1_GY_2 | 0.17 | 0.75 | 175.98 | 0.001 | Anthesis |
|  |  |  | Anth_2_a_2_GA_2_GY_2 | 0.20 | 0.73 | 176.32 | 0.000 | Grain filling |
|  | Rainfed | 2017 | CT_1_NDVI_1_GY_2 | 0.63 | 0.36 | 69.94 | 0.000 | Anthesis |
|  |  |  | CT_1_CCI_2_GY_2 | 0.62 | 0.37 | 71.85 | 0.000 | Both |
|  |  |  | CT_2_CCI_2_GY_2 | 0.52 | 0.41 | 88.25 | 0.000 | Grain filling |
|  |  | 2018 | NDVI_1_WBI_1_GY_2 | 0.57 | 0.41 | 90.34 | 0.000 | Anthesis |
|  |  |  | NDVI_1_WBI_1_TCARI_2_GY_2 | 0.57 | 0.41 | 93.66 | 0.000 | Both |
|  |  |  | CCI_2_TCARI_2_GY_2 | 0.51 | 0.44 | 100.34 | 0.000 | Grain filling |
|  |  | 2019 | GA_1_CT_2_a_2_GY_2 | 0.30 | 0.28 | 29.50 | 0.000 | Both |
|  |  |  | GA_1_GY_2 | 0.16 | 0.31 | 32.74 | 0.003 | Anthesis |
|  |  |  | a_2_GY_2 | 0.01 | 0.34 | 58.57 | 0.160 | Grain filling |
|  | Late | 2017 | a_2_CCI_2_GY_2 | 0.61 | 0.43 | 94.78 | 0.000 | Grain filling |
|  |  |  | TCARIOSAVI_1_CCI_2_GY_2 | 0.61 | 0.43 | 94.81 | 0.000 | Both |
|  |  |  | GA_1_TCARIOSAVI_1_GY_2 | 0.52 | 0.48 | 108.98 | 0.000 | Anthesis |
|  |  | 2018 | Flav_2_GY_2 | 0.26 | 0.64 | 151.13 | 0.000 | Grain filling |
|  |  |  | TCARIOSAVI_1_Flav_2_GY_2 | 0.27 | 0.63 | 152.94 | 0.000 | Both |
|  |  |  | Flav_1_TCARIOSAVI_1_GY_2 | 0.07 | 0.72 | 170.53 | 0.030 | Anthesis |
|  |  | 2019 | a_2_GA_2_GY_2 | 0.25 | 0.67 | 159.72 | 0.000 | Grain filling |
|  |  |  | a_1_a_2_GA_2_GY_2 | 0.24 | 0.67 | 163.54 | 0.000 | Both |
|  |  |  | GA_1_GY_2 | 0.11 | 0.72 | 168.55 | 0.003 | Anthesis |
| Coria | Rainfed | 2017 | WBI_1_CRI2_2_GY_2 | 0.19 | 0.98 | 207.33 | 0.000 | Both |
|  |  |  | WBI_1_GY_2 | 0.12 | 1.02 | 209.46 | 0.002 | Anthesis |
|  |  |  | GA_2_GY_2 | 0.09 | 1.04 | 211.78 | 0.006 | Grain filling |
|  |  | 2018 | NDVI_1_GY_2 | 0.09 | 0.65 | 147.73 | 0.008 | Anthesis |
|  |  |  | NDVI_1_TCARIOSAVI_2_GY_2 | 0.08 | 0.66 | 151.34 | 0.024 | Both |
|  |  |  | GA_2_GY_2 | 0.03 | 0.67 | 152.04 | 0.095 | Grain filling |
|  |  | 2019 | a_1_GA_2_GY_2 | 0.28 | 0.60 | 140.17 | 0.000 | Both |
|  |  |  | GA_1_GY_2 | 0.22 | 0.63 | 142.15 | 0.000 | Anthesis |
|  |  |  | a_2_GY_2 | 0.21 | 0.63 | 143.18 | 0.000 | Grain filling |
| Valladolid | Irrigation | 2017 | a_2_CCI_2_GY_2 | 0.75 | 0.53 | 121.43 | 0.000 | Grain filling |
|  |  |  | GA_1_a_2_CCI_2_GY_2 | 0.75 | 0.53 | 124.21 | 0.000 | Both |
|  |  |  | CT_1_GA_1_GY_2 | 0.38 | 0.82 | 182.33 | 0.000 | Anthesis |
|  |  | 2018 | Flav_1_NDVI_1_GY_2 | 0.51 | 0.52 | 122.86 | 0.000 | Anthesis |
|  |  |  | Flav_1_NDVI_1_NDVI_2_GY_2 | 0.53 | 0.50 | 122.94 | 0.000 | Both |
|  |  |  | NDVI_2_GY_2 | 0.42 | 0.56 | 131.73 | 0.000 | Grain filling |
|  |  | 2019 | GA_2_GY_2 | 0.13 | 1.37 | 260.89 | 0.001 | Grain filling |
|  |  |  | a_1_GA_2_GY_2 | 0.12 | 1.38 | 264.82 | 0.004 | Both |
|  |  |  | a_1_GY_2 | 0.03 | 1.46 | 269.27 | 0.092 | Anthesis |
|  | Rainfed | 2017 | CT_1_a_2_GY_2 | 0.25 | 0.97 | 204.77 | 0.000 | Both |
|  |  |  | a_2_GY_2 | 0.17 | 1.01 | 208.41 | 0.000 | Grain filling |
|  |  |  | TCARIOSAVI_1_GY_2 | 0.09 | 1.06 | 214.38 | 0.007 | Anthesis |
|  |  | 2018 | GA_2_PRI_2_GY_2 | 0.64 | 0.66 | 157.67 | 0.000 | Grain filling |
|  |  |  | PRI_1_GA_2_PRI_2_GY_2 | 0.64 | 0.66 | 161.04 | 0.000 | Both |
|  |  |  | NDVI_1_GY_2 | 0.51 | 0.77 | 177.25 | 0.000 | Anthesis |
|  |  | 2019 | NDVI_1_a_2_GY_2 | 0.45 | 0.54 | 130.32 | 0.000 | Both |
|  |  |  | a_2_GY_2 | 0.41 | 0.56 | 131.22 | 0.000 | Grain filling |
|  |  |  | NDVI_1_GY_2 | 0.37 | 0.58 | 136.90 | 0.000 | Anthesis |
|  | Late | 2017 | a_2_CCI_2_GY_2 | 0.61 | 0.43 | 94.78 | 0.000 | Grain filling |
|  |  |  | TCARIOSAVI_1_CCI_2_GY_2 | 0.61 | 0.43 | 94.81 | 0.000 | Both |
|  |  |  | CT_1_GA_1_CCI_2_GY_2 | 0.57 | 0.45 | 103.91 | 0.000 | Anthesis |

**Supplemental table 5**. Determination coefficients of the Pearson correlations between the carbon isotope composition and grain yield (GY). R^2^, coefficient of determination.

| **Location** | **Treatment** | **Year** | **R^2^** |
| --- | --- | --- | --- |
| Coria | Rainfed | 2018 | 0.023 |
|  |  | 2019 | 0.007 |
| Aranjuez | Irrigation | 2018 | 0.266 |
|  |  | 2019 | 0.125 |
|  | Rainfed | 2018 | 0.042 |
|  |  | 2019 | 0.176 |
|  | Late-planting | 2018 | 0.259 |
|  |  | 2019 | 0.003 |
| Valladolid | Irrigation | 2018 | 0.007 |
|  |  | 2019 | 0.034 |
|  | Rainfed | 2018 | 0.078 |
|  |  | 2019 | 0.006 |

# Supplementary Figures


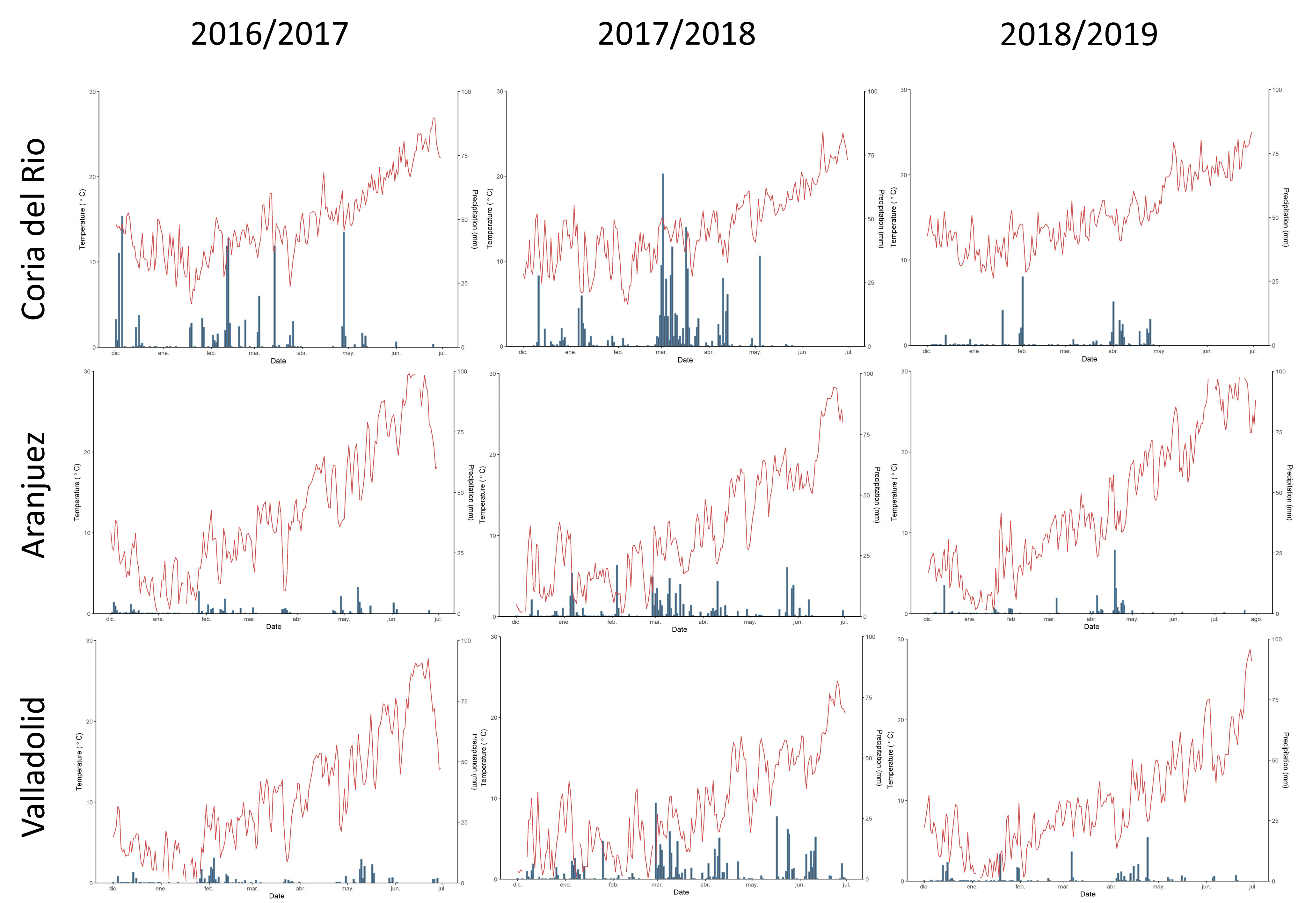


**Supplementary Figure 1.** Mean temperature (red) and rainfall (blue) in Coria del Rio, Aranjuez and Valladolid for the 2016/2017, 2017/2018 and 2018/2019 crop seasons.
